# Digital Activity Markers in Chronic Inflammatory Demyelinating Polyneuropathy

**DOI:** 10.1002/acn3.70137

**Published:** 2025-07-09

**Authors:** Lars Masanneck, Jan Voth, Noëmi Gmahl, Konstantin Jendretzky, Niklas Huntemann, Noah M. Werner, Linea Schmidt, Menekse Oeztuerk, Paula Quint, Christina B. Schroeter, Hans Peter Hartung, Thomas Skripuletz, Gerd Meyer zu Hörste, Tobias Ruck, Sven G. Meuth, Marc Pawlitzki

**Affiliations:** ^1^ Department of Neurology University Hospital and Medical Faculty University Hospital Düsseldorf Düsseldorf Germany; ^2^ Hasso Plattner Institute, Digital Engineering Faculty University of Potsdam Potsdam Germany; ^3^ Department of Neurology With Institute of Translational Neurology University Hospital Münster Münster Germany; ^4^ Department of Neurology Hannover Medical School Hannover Germany; ^5^ Brain and Mind Center, University of Sydney Sydney New South Wales Australia; ^6^ Department of Neurology Palacky University Olomouc Olomouc Czech Republic

**Keywords:** CIDP, neuromuscular disease, wearables

## Abstract

**Objective:**

To evaluate the utility of smartwatch and smartphone‐based activity metrics for assessing disease severity and quality of life in patients with chronic inflammatory demyelinating polyneuropathy (CIDP).

**Methods:**

In the electronic monitoring of disease activity in patients with CIDP (EMDA‐CIDP) trial, we performed a prospective observational study from January 2023 to July 2024 at university hospitals in Düsseldorf and Münster, with an independent validation cohort in Hannover. Eligible participants were adults with CIDP on stable intravenous immunoglobulin (IVIG) therapy. Clinical evaluations included established disability scales (I‐RODS and INCAT) and quality of life assessments. Activity metrics were captured via consumer‐grade smartwatches, with adherence criteria applied to ensure data quality. A real‐world smartphone‐based cohort of 20 patients was used as a comparator.

**Results:**

Among 46 participants (median age 64 Years [IQR 57–69]; 24% female), smartwatch‐derived maximum daily‐step count emerged as a robust indicator of disease severity. In 43 patients meeting wearable adherence criteria, maximum daily steps showed strong correlations with clinical scores, positively with I‐RODS (Spearman's *ρ* = 0.74) and inversely with INCAT (Spearman's *ρ* = −0.54). Additional smartwatch metrics correlated with quality of life domains, whereas smartphone‐derived metrics of a validation cohort exhibited weaker correlations.

**Interpretation:**

These results indicate that smartwatches many patients already use can yield valuable, objective data for assessing disease status in CIDP. Integrating smartwatch‐derived metrics into clinical assessments may enhance traditional evaluations and deepen understanding of disease progression and patient quality of life. These promising results warrant additional, larger‐scale studies in the future.

## 1 Introduction

Chronic inflammatory demyelinating polyneuropathy (CIDP) is a rare, immune‐mediated neuropathy characterized by progressive or relapsing symptoms lasting over 8 weeks [1]. Common CIDP symptoms include symmetric muscle weakness, dysesthesia, paresthesia, neuropathic pain, gait disturbances, and a reduction or loss of deep tendon reflexes [2]. To date, there are no treatments that can cure CIDP. Thus, long‐term therapy is usually required and includes classical immunosuppressants [3], corticosteroids, as well as subcutaneously [4] or intravenously administered immunoglobulins (IVIG) [5].

Because of CIDP's variability and fluctuating nature, effective monitoring is complex, often requiring both subjective assessments and objective instruments for accurate tracking [6]. Commonly used scales include the physician‐scored Inflammatory Neuropathy Cause and Treatment (INCAT) [5] scale and the self‐reported Inflammatory Rasch‐Built Overall Disability Scale (I‐RODS) [7, 8]. Manual muscle testing with Medical Research Council (MRC) grading [9] or grip strength assessments [10, 11] primarily captures the muscular weakness. However, clinical follow‐ups often miss subtle changes and are rater‐dependent [9, 12]. Repeated nerve conduction studies frequently fail to accurately assess disease activity, showing varying correlations with clinical progression [13]. Similarly, the usefulness of modern imaging techniques such as ultrasonography [14] or MRI for monitoring CIDP remains unclear [15] and is only accessible at specialized centers [16]. Lastly, blood biomarkers are under investigation, but have not yet proven reliable enough to guide current treatment decisions [17, 18].

Given these limitations, there is a critical need for new assessments to monitor disease activity. Digital Health Technologies (DHTs) such as connected wearables are increasingly used in neurology trials, offering continuous real‐life data on movement, sleep, and cognition [19].

As these measurements promise a novel dynamic and continuous evaluation of the patient's status, first digital assessments have now been endorsed by a regulatory body for inclusion in clinical trials. One such DHT measuring gait data in Duchenne muscular dystrophy showcases the potential in neuromuscular assessments [20]. Parametrizing neurological disorders using DHTs is further driven by efforts such as Mobilize‐D, a consortium that validated metrics across indications [21]. Meanwhile, simpler and widely accessible activity metrics, such as step count, are known to correlate with overall health, all‐cause mortality [22], and clinical outcomes in neurological diseases like multiple sclerosis [23]. With smartwatches offering relatively accurate step tracking [24] and becoming increasingly common [25], it is crucial to evaluate the potential of this data in the context of CIDP.

**FIGURE 1:**
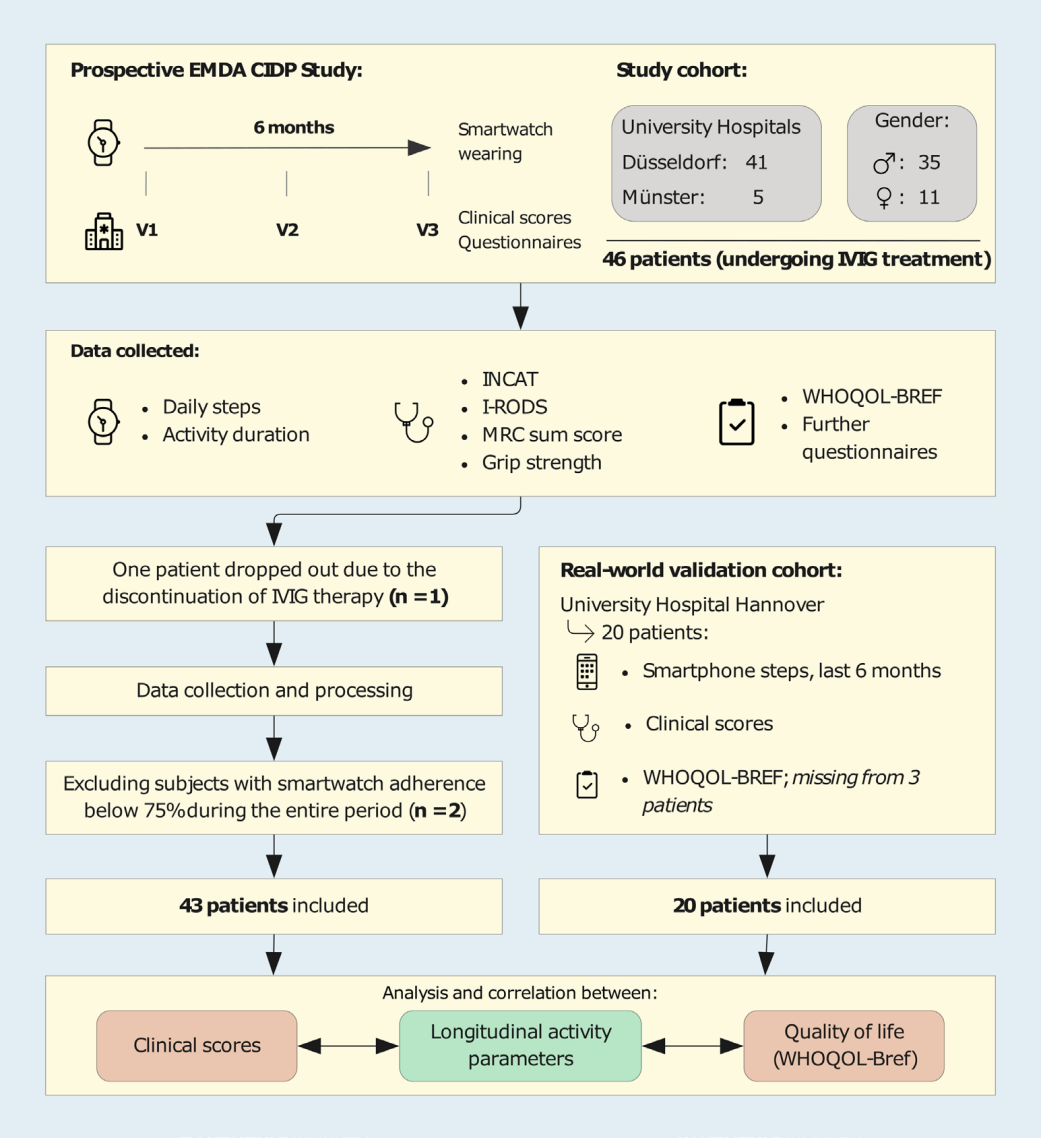
Study and analysis overview. This figure illustrates the design and process of the EMDA‐CIDP study and its here presented analysis, including patient recruitment, data collection, and analysis. The study followed 46 patients undergoing IVIG treatment at two university hospitals over 6 months, collecting various clinical scores and smartwatch‐derived activity data. After excluding one patient due to therapy discontinuation and two others for low smartwatch adherence, 43 patients were included in the final analysis. The study then validated findings using smartphone data from a real‐world cohort of 20 CIDP patients from University Hospital Hannover. The here presented analysis focused on correlating different intervals of wearable or smartphone‐derived activity parameters with clinical scores and QOL results. CIDP, Chronic Inflammatory Demyelinating Polyradiculoneuropathy; INCAT, Inflammatory Neuropathy Cause and Treatment; I‐RODS, Inflammatory Rasch‐built Overall Disability Scale; IVIG, Intravenous Immunoglobulin; MRC, Medical Research Council; WHOQOL‐Bref, World Health Organization Quality of Life‐BREF.

In this two‐center Electronic Monitoring of Disease Activity in Patients with CIDP (EMDA‐CIDP) trial [26], patients were monitored clinically and digitally using smartwatches for 6 months. Our findings indicate that simple smartwatch‐based motor assessments highly correlate with CIDP disease status and quality of life (QOL). Less precise, yet still valuable, similar metrics can be more easily obtained through patient smartphones, as shown in an independent validation cohort (Figure 1).

## 2 Subjects/Materials and Methods

### 2.1 Study Design

In the EMDA‐CIDP trial (NCT05723848) [26] CIDP patients on stable IVIG therapy were recruited from January 2023 to April 2024 at the University Hospitals of Düsseldorf and Münster, Germany. Patients were equipped with a smartwatch (Scanwatch, Withings, Paris, France) and were clinically, digitally, and serologically followed up for 6 months or the nearest scheduled visit. This study was conducted and reported in accordance with STROBE guidelines.

Inclusion required 2021 EAN/PNS CIDP criteria [2] (which was changed from the 2010 criteria in the initial protocol) [27], documented evidence of positive response to IVIG, ≥ 8 weeks of stable IVIG treatment and exclusion of CIDP variants. After written consent, patients received a smartwatch paired with their smartphone for data transmission. Patients were instructed to wear the smartwatch day and night, with the watch recording daily steps as well as the estimated duration of soft (renamed to light), moderate, and intense (or vigorous) activity (in seconds) according to The Physical Activity Guidelines for Americans [28] and the Compendium of Physical Activities [29].

Clinical scores (INCAT [4], I‐RODS [7, 8] and MRC‐sumscore [9], grip strength [10, 11], for details see Data S1) were recorded at baseline (V1), 3 months (V2), and 6 months (V3). An untransformed version of I‐RODS (raw questionnaire scores) was used throughout this work. Correlations with smartwatch data were based on the end‐of‐study (V3) outcomes. Various questionnaires were administered, including the Abbreviated World Health Organization Quality of Life (WHOQOL‐BREF) [30], which captures QOL across four domains (social, environmental, physical, mental) and has been used in similar neurological settings [30, 31].

### 2.2 Validation Cohort

At Hannover Medical School's CIDP center, patients received routine treatment with clinical scoring, completed WHOQOL‐BREF, and reported retrospective average and maximum smartphone step counts, as recorded by their technological platform (e.g., Apple Health). Smartphone data from the original smartwatch cohort was unavailable due to integration with smartwatch data.

### 2.3 Adherence Criteria

For activity analysis, adherence was defined as 75% of wearing time for wake hours (6:00 A.M. to 11:00 P.M.). Days below this threshold were excluded. Patients failing adherence on > 25% of days were excluded. Rarely, data sync issues led to < 1% missed activity, affecting 5 users. These intervals were marked as not worn. See Data S1 for details.

### 2.4 Technical, Correlation and Regression Analyses

For normality assessment and distribution statistics see Data S1. Normal data are reported as mean with standard deviation (SD); median is reported with interquartile range (IQR).

Pearson or Spearman's correlation was used as appropriate (see Data S1), with correlation coefficients interpreted according to Chan [32]. Bootstrapped 95% confidence intervals (CIs) for selected correlation coefficients were calculated using 1000 resampled datasets.

For exploratory hypotheses generation involving multiple comparisons, the Benjamini‐Hochberg procedure was used to control the false discovery rate. For more stringent hypothesis testing of correlations, Holm correction was used.

To approximate the robustness of maximum steps of varying sampling timeframes, we extracted all possible consecutive intervals of specified lengths (7, 14, 30, and 60 days) from the steps data and calculated the Intraclass Correlation Coefficient (ICC) following *Woelfle and colleagues* [33]. ICC calculation was interpreted following Cicchetti [34].

Age and body mass index (BMI) influence on maximum steps predicting I‐RODS was tested via Ridge regression with polynomial features and z‐score normalization. 5‐fold cross‐validation was applied (Data S1 for details).

Linear regression was used to create visual trendlines to illustrate the relationship between maximum steps per day and selected clinical parameters, despite potential violations of assumptions. For more accurate representations of these relationships, non‐parametric LOESS (Locally Estimated Scatterplot Smoothing) regression was additionally visualized.

### 2.5 Software

All analyses used Python 3.8.15 (Python Software Foundation, Delaware, USA). See Data S1 for packages.

## 3 Ethical Considerations

Approval was obtained from Ethics Committees in Düsseldorf (No. 2022‐1881_1), Münster (No. 2023‐106‐b‐S), and Hannover (No. 9741_BO_S_2021). All participants provided informed consent, and all trial activities were in accordance with the Declaration of Helsinki.

## 4 Results

### 4.1 Cohort Characteristics

Forty‐six patients were recruited across centers, with the majority (41) enrolled in Düsseldorf. One patient discontinued participation due to cessation of IVIG therapy. The median age at inclusion was 64 (IQR: 57–69) years, with a median disease duration of 5 (IQR: 2–10) years. Most patients (75.6%) were male. Baseline clinical scores were a median INCAT of 2 (IQR: 1–4) and a median untransformed I‐RODS of 40 (IQR: 32–45) (see Table 1).

**TABLE 1:** Trial Cohort Characteristics.

*n* (%)		0/46	46 (100)
Study center, *n* (%)	MS	0/46	5 (11)
	DD		41 (89)
Participant study dropout, *n* (%)		0/46	1 (2)
Sex, *n* (%)	w	0/45	11 (24)
	m		34 (76)
Age at inclusion [y], median [Q1, Q3]		0/45	64 [57, 69]
Age at diagnosis [y], median [Q1, Q3]		0/45	59 [51, 65]
Disease duration at inclusion [y], median [Q1, Q3]		0/45	5 [2, 10]
Baseline INCAT Disability Score, median [Q1, Q3]		0/45	2 [1, 4]
Baseline untransformed I‐RODS, median [Q1, Q3]		0/45	40 [32, 45]
Baseline MRC‐sumscore, median [Q1, Q3]		0/45	55 [50, 59]
V3 INCAT Disability Score, median [Q1, Q3]		0/45	3 [1, 4]
V3 untransformed I‐RODS, median [Q1, Q3]		0/45	40 [28, 43]
V3 MRC‐sumscore, median [Q1, Q3]		0/45	54 [49, 60]
Duration of immunoglobulin therapy at inclusion [mo], median [Q1, Q3]		0/45	23 [9, 85]
Immunoglobulin treatment interval [d], median [Q1, Q3]		0/45	28 [28, 42]
BMI (kg/m^2^), median [Q1, Q3]		0/45	27.3 [25, 30]
Duration in study [d], median [Q1, Q3]		0/45	176 [168, 203]
% of hours with data from digital device per subject, median [Q1, Q3]		0/45	98.0 [94.4, 99.1]
% days meeting digital adherence target per subject, median [Q1, Q3]		0/45	97.6 [93.6, 99.3]
Daily wearable adherence target for activity analysis met, *n* (%)		0/45	43 (96)
Average steps per day per subject, mean (SD)		0/43	4222 (2391)
Median steps per day per subject, median [Q1, Q3]		0/43	3818 [3000, 5065]
Maximum steps per day per subject, mean (SD)		0/43	12,280 (6125)

Abbreviations: BMI, body mass index; d, day; DD, University Hospital Düsseldorf; I‐RODS, Inflammatory Rasch‐built Overall Disability Scale; INCAT, Inflammatory Neuropathy Cause and Treatment; m, men; mo, month; MRC, Medical Research Council; MS, University Hospital Münster; *n*, quantity; Q1, lower quartile; Q3, upper quartile; SD, standard deviation; V3, visit 3 (end‐of‐study visit); w, women; y, year.

Regarding activity, the cohort (43 patients meeting wearable adherence criteria) averaged 4222 (SD: 2391) steps per day. Over the entire observation period, an average daily maximum of 12,280 (SD: 6125) steps was recorded. Patients showed a median of 10.0 (IQR 2.5–29.0) daily minutes of combined moderate‐vigorous activity, for many likely below the weekly recommendation of 150–300 min for adults [28]. More detailed distributions of underlying data are illustrated in Figure S1. Overall, the cohort did not show meaningful progression in the 6‐month observation period, with clinical values showing no significant difference between V1 and V3.

The real‐world smartphone validation cohort from Hannover included 20 patients with similar disease severity, with neither age nor disease severity differing significantly from the original cohort. On average, smartphone data indicated that patients walked 4546 steps per day (SD: 3380) (Table S1 for validation cohort statistics).

### 4.2 Smartwatch Adherence

Over the entire study duration, patient adherence of wearing the smartwatch was high, with a median of 98.0% (IQR: 94.4%–99.1%) of total hours, translating to a median of 97.6% (IQR: 93.6%–99.3%) days per patient meeting the predefined daily adherence target. A summary of adherence per user is illustrated in Figure S2 with detailed information listed in Table S2.

### 4.3 Correlation Activity Metrics With Clinical and QOL Parameters

Correlation analyses of smartwatch activity parameters with clinical scores identified steps and moderate activity as the most promising concepts, prompting further analyses focused on these two metrics (Figure S3).

**FIGURE 2:**
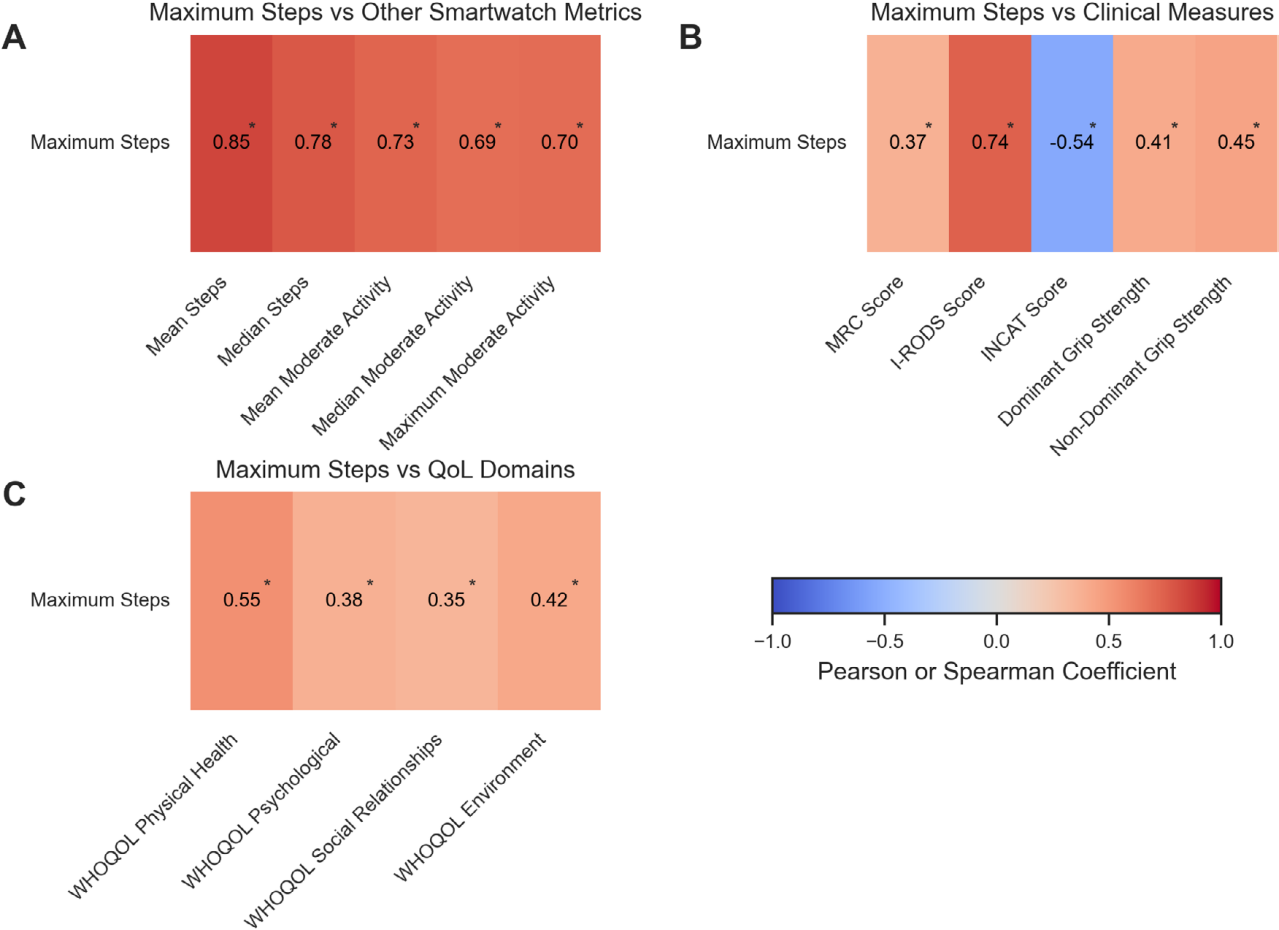
Targeted correlation heatmap for maximum daily‐step count in CIDP. (A) Correlation between maximum steps and other smartwatch‐derived activity metrics (mean, median, and moderate activity aggregates). (B) Correlation between maximum steps and clinical disability measures (MRC‐sumscore, I‐RODS, INCAT) together with dominant and non‐dominant grip strength. (C) Correlation between maximum steps and the four quality‐of‐life domains of the WHOQOL‐BREF. Red indicates positive correlations, while blue indicates negative correlations. Stars denote statistically significant correlations after applying either the more conservative Holm correction (A) or the FDR Benjamini‐Hochberg correction for hypothesis generation (B, C) across multiple comparisons. The *p* value adjustments use the full correlation matrix shown in Figure S4; therefore, the adjusted thresholds applied here are conservative relative to this reduced presentation. Pearson or Spearman correlation coefficients were used based on data distribution. All unadjusted and adjusted *p* values as well as confidence interval estimates can also be found alongside the correlation and correction method used for each pair in Table S2. Figures S5 and S6 provide confidence interval estimates for some of these correlations. CIDP, Chronic Inflammatory Demyelinating Polyradiculoneuropathy; FDR, False Discovery Rate; I‐RODS, Inflammatory Rasch‐built Overall Disability Scale; INCAT, Inflammatory Neuropathy Cause and Treatment; MRC, Medical Research Council; QOL, Quality of Life; WHOQOL‐BREF, World Health Organization Quality of Life‐BREF.

Aggregated metrics (mean, median, maximum) over the entire trial of both steps and moderate activity showed moderate‐to‐very strong intercorrelations of smartwatch‐derived variables (Figure S4A), indicating that while related, they do not capture identical aspects of activity. When correlated with clinical scores and WHOQOL‐BREF domains, analyses revealed moderate‐to‐strong associations with I‐RODS (all positive) and INCAT (all negative) and varying significant fair to moderately strong correlations with all QOL domains (see Figure S4B,C). Maximum steps appeared as the most promising marker as it had the strongest correlation with I‐RODS (0.74) and the most negative with INCAT (−0.54), consistent with the clinical interpretation that I‐RODS decreases with disease severity (and presumably lower activity), whereas INCAT increases. Maximum steps additionally had significant correlations with all QOL domains (see Figure 2). For details on coefficients, see Table S3 and bootstrapped CIs in Figures S5 and S6. Given the best correlation results of maximum daily steps, its simplicity and the ease of measurement across various tools, we further focused on maximum steps.

### 4.4 Evaluation of Maximum Daily Steps as Disease Severity Marker

Building on its strong ties with clinical scores and QOL measures, we aimed to establish [1] which daily‐step quantile best reflects CIDP severity and [2] the minimal monitoring duration needed for a reliable “maximal steps” estimate.

**FIGURE 3:**
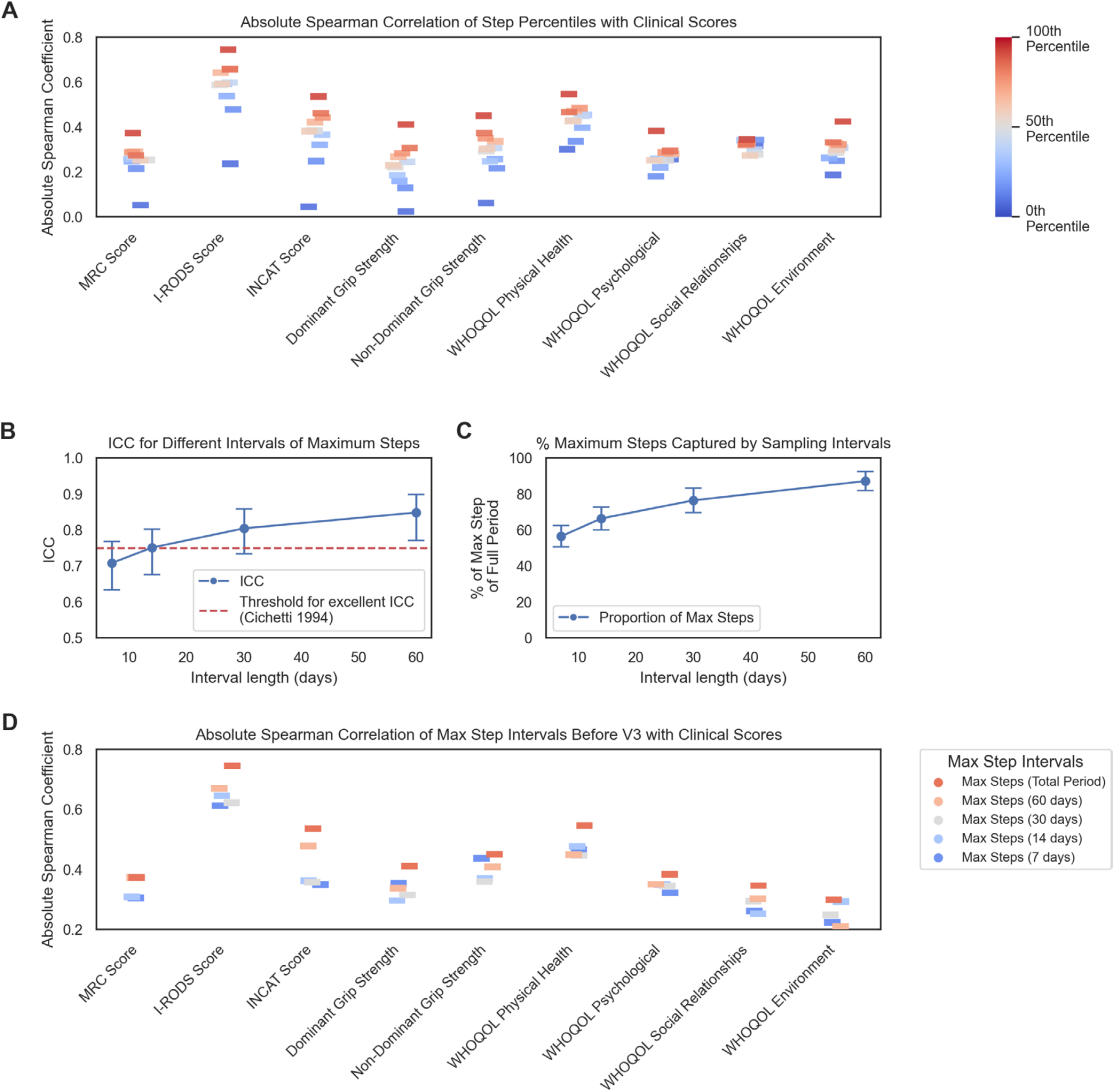
Evaluating maximum steps as a robust step parameter for correlation with CIDP severity and quality of life metrics. This figure examines the efficacy of maximum steps as a parameter for assessing the severity of CIDP and the appropriate timeframe required for meaningful correlations with clinical scores and QOL results. (A) The strip plot presents the absolute (non‐negative) Spearman correlations of different step percentiles (in steps of 10), including maximum steps (darkest red) and other percentiles, with various clinical CIDP parameters (e.g., I‐RODS or INCAT Score) and QOL domains (e.g., WHOQOL Physical Health). The correlation values are represented as rectangles on the plot, with higher percentiles indicated in red and lower percentiles in blue. Figure S7 provides a detailed heatmap of these correlations, including statistical significances. Generally, maximum steps appear to have comparably high correlations. (B) The Intraclass Correlation Coefficient (ICC) is plotted with estimated 95% confidence intervals for various intervals of maximum steps, assessing the stability of this parameter over different timeframes. Shorter intervals for evaluating maximum steps generally showed a lower ICC. The red horizontal line marks the threshold for “excellent” ICC (≥ 0.75), as defined by Cicchetti. (C) This lineplot shows the proportion of maximum steps captured by different intervals (measured as the last X days before the study end) expressed as a percentage, along with 95% confidence intervals. This analysis helps determine how well shorter intervals reflect overall maximum step data. (D) The correlation of maximum steps from different intervals (7, 14, 30, and 60 days) with clinical and QOL parameters is depicted in a strip plot. Strip plots were used to improve the visibility of data points that might otherwise overlap, enhancing the interpretation of how different timeframes correlate with CIDP severity and QOL outcomes. CIDP, Chronic Inflammatory Demyelinating Polyradiculoneuropathy; ICC, Intraclass Correlation Coefficient; INCAT, Inflammatory Neuropathy Cause and Treatment; I‐RODS, Inflammatory Rasch‐built Overall Disability Scale; Max, Maximum; QOL, Quality of Life; WHOQOL, World Health Organization Quality of Life.

To answer these, we ran exploratory correlations of different step quantiles against clinical and WHOQOL‐BREF outcomes (Figure 3A; Figures S7–S9). Maximum daily steps had the strongest correlations despite potential age/BMI confounding. In contrast, correlations between the previously second‐best correlating activity metric, moderate activity, were more variable and more heterogeneous, reinforcing our previous decision to prioritize maximum steps.

Next, we assessed reliability and coverage by computing ICCs and capture‐rate percentages for different sampling windows. ICCs rose with interval length, indicating more stable estimates as short‐term fluctuations average out. Notably, even a 14‐day window yielded an ICC of 0.75 (95% CI 0.67–0.81), already considered “excellent” reliability [34], rising to 0.85 (95% CI 0.77–0.90) over 60 days (Figure 3B). As anticipated, the proportion of absolute maximum daily steps, expressed as a share of the total maximum captured steps during the entire observational period, increased with longer measurement intervals up to 87.2% (95% CI: 81.9–92.5) for 60‐day intervals. While the ICCs indicate stable measurements, these proportions show that short intervals can already approximate the maximum daily steps for the ~6‐month observational period (Figure 3C). Longer intervals tended to strengthen correlations, though these trends varied by outcome and remain exploratory (Figure 3D). Taken together, these findings suggest that maximum daily steps, across varying intervals, may be a reliable parameter for assessing CIDP severity and its correlation with QOL metrics.

### 4.5 Association Patterns of Clinical and Digital Measurements

After establishing maximum daily steps as a reliable marker, we applied ridge regression (accounting for age and BMI) and confirmed a strong positive association between maximum steps and I‐RODS, with negative coefficients for age and BMI. Including polynomial terms revealed a leveling‐off effect at high step counts, suggesting a plateau in the correlation between maximum daily steps and I‐RODS (Figure S10).

**FIGURE 4:**
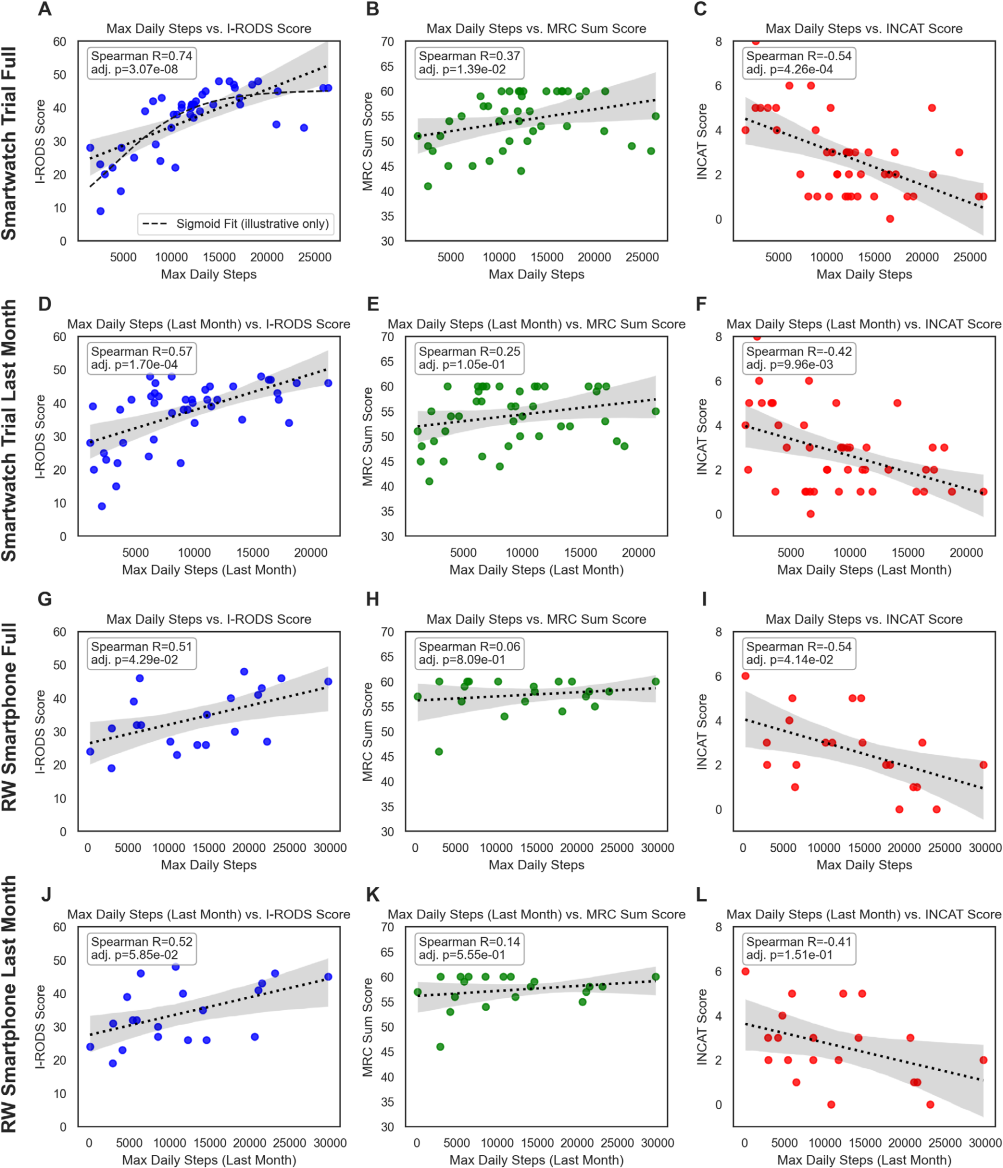
Scatter plots of maximum daily steps correlated with clinical scores in CIDP patients and a real‐world cohort. This figure presents scatter plots illustrating the relationship between maximum daily steps and three clinical scores: Untransformed I‐RODS (blue—left column, theoretical maximum 48), MRC‐sumscore (green—middle column, theoretical maximum 60), and INCAT Score (red—right column, theoretical maximum 10). The analysis is conducted across different observation periods and cohorts. All reported R values in the graphs are row‐wise Holm‐adjusted Spearman correlation coefficients, reflecting the rank‐based relationship between maximum steps and the clinical scores. The linear trendlines in these plots are for illustrative purposes only and should be interpreted as approximations, not as precise representations of the underlying relationships, especially given the ordinal nature of the MRC and INCAT scores. For a technically more accurate representation of the relationships, non‐parametric LOESS regression trendlines are provided in Figure S11. (A–C) display the correlations for the EMDA‐CIDP patients' (*n* = 43) maximum daily steps over the entire observational period. Notably, in panel (A), a fitted sigmoid function is included to illustrate a possible leveling‐off effect at higher step counts, suggesting a potential ceiling effect in the relationship between maximum steps and untransformed I‐RODS scores. (D–F) show the correlations for the maximum daily steps during the last month of the EMDA‐CIDP study period. (G–I) illustrate the correlations for maximum daily steps over a 6‐month period in the control real‐world smartphone cohort (*n* = 20). (J–L) present the correlations for the last month's maximum daily steps within the same real‐world cohort. Overall, these plots demonstrate varying degrees of correlation between maximum daily steps and clinical severity, with generally similar trends between the EMDA‐CIDP study cohort and the real‐world control cohort. The strongest correlations are observed with I‐RODS and INCAT scores, especially in the EMDA cohort, while correlations with the MRC‐sumscore are generally weaker. CIDP, Chronic Inflammatory Demyelinating Polyradiculoneuropathy; EMDA, Electronic Monitoring of Disease Activity; INCAT, Inflammatory Neuropathy Cause and Treatment; I‐RODS, Inflammatory Rasch‐built Overall Disability Scale; LOESS, Locally Estimated Scatterplot Smoothing; MRC, Medical Research Council; RW, Real‐World.

When visualized, this association of maximum daily steps and untransformed I‐RODS appears as a sigmoid curve, with the positive correlation (*R* = 0.74, *p* < 0.0001) flattening between 10,000 and 15,000 steps (Figure 4A), supporting the aforementioned assumption. Similar trends were observed for MRC‐sumscore (*R* = 0.37, *p* = 0.0139) with a weak leveling‐off and a clear negative correlation with INCAT (*R* = ‐0.54, *p* = 0.0004) (Figure 4B,C). The leveling‐off effect is particularly evident in I‐RODS and INCAT when using a LOESS trendline, a local regression method that smooths data points, rather than a linear trendline (Figure S11). For I‐RODS, this may also be partly explained by a ceiling effect, as the scale has a maximum of 48 points. Even using only the last month's data, these relationships persisted, though correlations weakened and the MRC correlation lost significance (Figure 4D–F).

In the validation cohort with retrospective smartphone data, 6‐month correlations remained significant for I‐RODS (*R* = 0.51, *p* = 0.0429) and INCAT (*R* = −0.54, *p* = 0.0414), but not with the last month's data, nor the leveling‐off pattern (Figure 4G–L, Figure S11). WHOQOL physical, psychological, and environmental domains correlated strongly with trial‐derived steps, but not with the smartphone data (Figures S12, S13). Hourly maximum step analysis revealed robust clinical correlations, though weaker than daily steps, while time‐of‐day analysis showed stronger correlations for afternoon/evening steps, and a notable positive link between mean afternoon and maximum nightly steps with the social WHOQOL domain (Figure S14).

## 5 Discussion

Despite advancements in the CIDP landscape, novel monitoring approaches for disease monitoring are still needed. The EMDA‐CIDP trial investigated whether readily‐available consumer‐grade smartwatch data could reflect clinical disease activity and QOL. Findings suggest that recording motor activity via these devices effectively captures disease status and may support more comprehensive monitoring in CIDP.

With the rising prevalence of wearable devices in the general population [25], patient‐collected data present a valuable opportunity to gain a more comprehensive understanding of patient health without imposing additional burdens, complex new assessments, or visits. In our study, parameters such as maximum daily steps (over a certain timeframe) provided insight into the disease activity and QOL. This metric is particularly suited to CIDP as it reflects a patient's physical potential, unlike mean or median measurements, which capture habitual activity that may not be limited by the disease to the same extent. This concept parallels the first digital endpoint recommended by European regulators, stride velocity at the 95th percentile in Duchenne muscular dystrophy [20], which also focuses on maximal physical capacity. While the correlation between maximum daily steps and clinical scores diminishes above 10,000 steps, it remains almost linear below this threshold, suggesting increased value in more affected patients. Moreover, our analyses demonstrate that the metric remains reliable even with data collected over shorter periods than 6 months. Based on the observed reliability and existing literature on fluctuations in daily activity [35], 1 to 2 months of monitoring is likely sufficient for meaningful assessments. This approach offers advantages over the single‐point evaluations in clinical practice, which are more vulnerable to day‐to‐day variability. Notably, adherence exceeded 95% over 6 months, highlighting patient openness to passive monitoring even at higher ages and contrasting dropout trends seen with frequent active assessments [36] Overall, passive activity monitoring holds promise for CIDP and aligns with positive results in other neurological disorders, including multiple sclerosis [23] or amyotrophic lateral sclerosis [37].

For the first time, we documented CIDP patients' daily activity levels, which often fall below recommended guidelines [28] and reach step counts linked to higher overall mortality in the general population [22]. Notably, the cohort comprised only CIDP patients considered stable under IVIG treatment with comparably low clinical affection, yet they still experienced likely disease‐related reductions in daily activity, an aspect not adequately addressed in current monitoring and treatment approaches. Moreover, reduced motor activity was correlated with declines across subdomains of the WHOQOL, highlighting the impact of CIDP and reduced activity potential on various aspects of patients' lives.

To assess whether smartphones, which are more readily available, could yield similar data, we analyzed an unrelated validation cohort. Analyses showed similar but weaker correlations, likely due to uncontrolled adherence and the limited suitability of smartphones for continuous tracking. For any technology measuring activity, it is essential to track wearing behavior to ensure data integrity—particularly with smartphones, which are not worn continuously like smartwatches. There nowadays is a clear gradient between the almost universally available smartphone, the less accessible but more accurate smartwatch, and highly specialized monitoring equipment, which can capture a wide range of neuromuscular functions and performs on par with or exceeds traditional neurological assessments [38, 39].

Overall, the study highlights the potential of unobtrusively collected digital data to inform disease status and potentially progression in clinical practice. Although integrating wearable data into healthcare systems is currently lacking, it is crucial for successful implementation. Nonetheless, these findings may encourage clinicians to ask about basic activity metrics, such as maximum daily steps over the past few months, which are accessible through most fitness tracker applications.

This study is subject to several limitations. While wrist‐worn sensors generally capture a broad range of activities, validation of our specific device is limited. However, comparisons within individuals should still be reliable, though absolute step counts may need caution. Furthermore, while we focused on quantitative changes, many neurological diseases also involve qualitative changes. Ongoing algorithm and consortium efforts may soon offer new metrics (e.g., step length) that could provide more precise insights [21]. Upper extremity symptoms might also be underrepresented, though clinical scores, partially measuring upper extremity function, aligned well with the activity data. Given the demonstrated influence of age and BMI, one has to be aware of the generally low specificity of step count, and normalization methods should be developed. Moreover, the smartphone cohort was small, uncontrolled for adherence, and used diverse devices, intentionally reflecting real‐world conditions.

In this proof‐of‐concept study, we did not observe significant clinical decline, likely due to the short observation period. Extending digital monitoring to longer periods is crucial to detect relapses and evaluate the predictive potential of digital tools more robustly. Subsequent analyses, incorporating data from other neurological diseases, may identify more complex “behaviorome” [40] patterns, cross‐disease composite biomarkers and implications for dose adjustment.

## Author Contributions

L.M., M.P. and S.G.M. conceived and designed the study. Study coordination and oversight were managed by L.M., M.P., S.G.M., T.R. and GmzH. Data collection and patient visits were conducted by L.M., J.V., N.G., N.H., N.M.W., M.O., P.Q. and C.B.S. K.J. and T.S. were responsible for data collection of the validation cohort. Data analysis was performed by L.M., M.P., J.V. and L.S. The initial manuscript draft was prepared by L.M., M.P. and J.V., and all authors contributed to manuscript revision and approved the final version. This study received no funding and was investigator‐initiated.

## Conflicts of Interest

L.M. reports no conflicts of interest related to this study. He has received honoraria for lecturing, consulting, and travel expenses for attending meetings from Biogen, Merck, Sanofi, argenX, Roche, Alexion, and Novartis, all outside the scope of this work. His research is funded by the German Multiple Sclerosis Foundation (DMSG) and the Deutsche Forschungsgemeinschaft (DFG, German Research Foundation)—493,659,010. J.V. reports no conflicts of interest. N.G. reports no conflicts of interest. K.J. reports no conflicts of interest related to this study. He has received research support from Else Kröner Fresenius Foundation and travel compensation and congress fee from Merck, Neuraxpharm and Novartis. N.H. reports personal fees from ArgenX, Merck, Novartis and Viatris; travel reimbursements and meeting attendance fees from Alexion, ArgenX, Merck, and Novartis; research support by the UKD FUTURE program of the Deutsche Forschungsgesellschaft outside the scope of this study. N.M.W. reports no conflicts of interest. L.S. reports no conflicts of interest. M.O. reports no conflicts of interest. P.Q. reports no conflicts of interest. C.B.S. reports no conflicts of interest. H.P.H. reports no conflicts of interest related to this study. He reports honoraria for lecturing and travel expenses for attending meetings from Novartis and Roche. T.S. reports no conflicts of interest related to this study. He reports research support from Alnylam Pharmaceuticals, CSL Behring, Novartis, Siemens; honoraria for lectures and travel expenses for attending meetings from Alexion, Alnylam Pharmaceuticals, argenx, Bayer Vital, Biogen, Bristol Myers Squibb, Celgene, Centogene, CSL Behring, Euroimmun, Grifols, Hexal AG, Horizon, Janssen‐Cilag, Merck Serono, Novartis, Pfizer, Roche, Sanofi, Siemens, Swedish Orphan Biovitrum, Teva, Viatris; consultant fees from Alexion, Alnylam Pharmaceuticals, Biogen, Centogene, CSL Behring, Grifols, Hexal AG, Janssen‐Cilag, Merck Serono, Novartis, Roche, Sanofi, Swedish Orphan Biovitrum, Viatris. GmzH reports no conflicts of interest related to this study. He was supported by grants from the Deutsche Forschungsgemeinschaft (DFG) (ME4050/12–1, ME4050/13–1) and from the Bundesministerium für Bildung und Forschung (BMBF) ‘Lipid Immune Neuropathy Consortium’. T.R. reports no conflicts of interest related to this study. S.G.M. reports no conflicts of interest related to this study. He has received honoraria for lecturing and travel expenses for attending meetings from Almirall, Amicus Therapeutics Germany, ArgenX, Bayer Health Care, Biogen, Celgene, Diamed, Genzyme, MedDay Pharmaceuticals, Merck Serono, Novartis, Neuraxpharm, Novo Nordisk, ONO Pharma, Roche, Sanofi‐Aventis, Chugai Pharma, QuintilesIMS, and Teva. His research is funded by the German Ministry for Education and Research (BMBF), Bundesinstitut für Risikobewertung (BfR), Deutsche Forschungsgemeinschaft (DFG), Else Kröner Fresenius Foundation, Gemeinsamer Bundesausschuss (G‐BA), German Academic Exchange Service, Hertie Foundation, Interdisciplinary Center for Clinical Studies (IZKF) Muenster, German Foundation Neurology, and by Alexion, Almirall, Amicus Therapeutics Germany, Biogen, Diamed, Fresenius Medical Care, Genzyme, HERZ Burgdorf, Merck Serono, Novartis, ONO Pharma, Roche, and Teva, all outside the scope of this study. M.P. reports no conflicts of interest related to this study. He has received honoraria for lecturing and travel expenses for attending meetings from Alexion, ArgenX, Bayer Health Care, Biogen, Hexal, Merck Serono, Neuraxpharm, Novartis, Roche, Sanofi‐Aventis, Takeda, and Teva. His research is funded by ArgenX, Biogen, Hexal, and Novartis, all outside the scope of this study.

## Supporting information


Data S1.

## Data Availability

The data that support the findings of this study are available on request from the corresponding author. The data are not publicly available due to privacy or ethical restrictions. Requests will be processed within six weeks and are subject to the data protection guidelines of University Hospitals Düsseldorf and Münster.
