# Supplementary material for: Digital Activity Markers in Chronic Inflammatory Demyelinating Polyneuropathy
Source: Ann Clin Transl Neurol. 2025 Jul 9;12(10):2045–55. doi: 10.1002/acn3.70137 (PMC12516250; doi:10.1002/acn3.70137)
Supplement: Supplementary file 1 — Data S1. [file ACN3-12-2045-s001.pdf]

Contents

Supplementary Methods 2
Supplementary References 3
Supplementary Table 1: Smartphone Real-World Cohort Characteristics 4
Supplementary Table 2: Adherence Data 5
Supplementary Table 3: Post-hoc Comparison Results 6
Supplementary Figure 1: Cohort overview of steps and moderate activity aggregation in the analyzed trial population (n=43) 9
Supplementary Figure 2: Smartwatch adherence among study participants 10
Supplementary Figure 3: Screening matrix of spearman correlation coefficients between smartwatch activity and clinical scores 11
Supplementary Figure 4: Bootstrapped spearman correlation coefficients of smartwatch parameters with clinical scores 12
Supplementary Figure 5: Bootstrapped spearman correlation coefficients of selected clinical and smartwatch variables with WHOQOL domains 14
Supplementary Figure 6: Heatmap of spearman correlations between percentiles of steps, clinical/QOL values, age, and BMI in CIDP patients 15
Supplementary Figure 7: Absolute spearman correlations of moderate activity percentiles with clinical scores and QOL results 16
Supplementary Figure 8: Heatmap of spearman correlations between percentiles of moderate activity, clinical/QOL values, age, and BMI in CIDP patients 17
Supplementary Figure 9: Ridge regression coefficients for I-RODS prediction in CIDP patients 18
Supplementary Figure 10: Scatter plots of maximum daily steps correlated with clinical scores in CIDP patients and a real-world cohort using LOESS regression 19
Supplementary Figure 11: Scatter plots of maximum daily steps correlated with WHOQOL domains in CIDP patients and a real-world cohort 21
Supplementary Figure 12: Scatter plots of maximum daily steps correlated with WHOQOL domains in CIDP patients and a real-world cohort using LOESS regression 23
Supplementary Figure 13: Heatmap of spearman correlations between daytime/peak steps and clinical/QOL metrics in CIDP patients 25

Supplementary Methods

Clinical scores

The INCAT[4] measures arm and leg disability on a 10-point scale, with each limb rated from 0 to 5. Leg disability is based on walking ability, while arm disability is assessed by tasks like handling small coins. A score of 0 indicates full functioning. The I-RODS [1,2] assesses 24 daily activities related to activity and social participation. Each activity item is scored from 0 (impossible) to 2 (easily performed), with a total score ranging from 0 to 48. The MRC-sumscore [3] assesses muscle function in six muscle groups of the upper and lower limbs: upper arm abductors, elbow flexors, wrist extensors, hip flexors, knee extensors, and foot dorsal flexors. Each group is scored from 0 (paralysis) to 5 (normal strength), with a total score ranging from 0 to 60. Grip strength [4,5] was measured using a Martin Vigorimeter. A squeezable rubber ball connected to a manometer provides a continuous measurement of grip strength, ranging from 0 to 160 kPa. The highest value from three consecutive measurements was recorded.

Smartwatch adherence calculation and criteria

Wake hours were set from 6:00 AM to 11:00 PM, based on the patients' documented sleep habits and adjusted for location/time zone. Only days that met these adherence criteria were included in the analysis. Patients who failed to meet the adherence criteria on more than 25% of the days were excluded from the study. In rare cases (<1% of hours or days) we observed that the watch was properly worn but activity data was not properly synchronized, most likely due to technical problems of watch and smartphone setup. This impacted 5 users, the affected days and hours were counted as not worn.

Normality assessment and distribution statistics

Normality was tested using both Shapiro-Wilk and D'Agostino-Pearson tests. Based on the normality results, paired t-tests or Wilcoxon signed-rank test were applied to compare baseline vs end-of-study data.

Correlation coefficients

For pairs of metric variables, Pearson correlation was utilized if both variables exhibited normal distribution and a linear relationship. For all other variable pairs and non-linear relationships, Spearman's correlation was used,

Software

Analyses were performed using Python 3.8.15 (Python Software Foundation, Delaware, USA) with the SciPy package version 1.9.3, Scikit-Learn package version 1.3.0 and the NumPy package version 1.23.5. The results were visualized using matplotlib version 3.6.2 with seaborn version 0.12.0. A web application to synchronize, organize and visualize data was setup based on the dash framework version 2.6.1.

Ridge regression

To assess potential influence and confounding potential of age and body mass index (BMI) on maximum steps prediction of I-RODS, we carried out a Ridge-regression with polynomial features of this sub dataset. Only for this regression, the underlying dataset underwent z-score normalization. To then capture potential non-linear relationships and interactions between variables, we generated second degree polynomial features. Ridge regression was employed as the primary modeling technique to manage multicollinearity and prevent overfitting. Due to the comparably small sample size of such an approach, we further validated the model's performance using 5-fold cross-validation, which involved splitting the data into five subsets, training on four, and testing on the fifth, rotating through all subsets. This process provided cross-validated estimates of Mean Squared Error (MSE) and R^2, along with an analysis of the stability of the regression coefficients across different folds.

Supplementary References

1. van Nes SI, Vanhoutte EK, van Doorn PA, et al. Rasch-built Overall Disability Scale (R-ODS) for immune-mediated peripheral neuropathies. *Neurology*. 2011;76(4):337-345. doi:10.1212/WNL.0b013e318208824b

2. Vanhoutte EK, Draak THP, Gorson KC, et al. Impairment measures versus inflammatory RODS in GBS and CIDP: a responsiveness comparison. *Journal of the Peripheral Nervous System*. 2015;20(3):289-295. doi:10.1111/jns.12118

3. Vanhoutte EK, Faber CG, van Nes SI, et al. Modifying the Medical Research Council grading system through Rasch analyses. *Brain*. 2012;135(5):1639-1649. doi:10.1093/brain/awr318

4. Draak THP, Gorson KC, Vanhoutte EK, et al. Correlation of the patient's reported outcome Inflammatory-RODS with an objective metric in immune-mediated neuropathies. *European Journal of Neurology*. 2016;23(7):1248-1253. doi:10.1111/ene.13025

5. Vanhoutte EK, Latov N, Deng C, et al. Vigorimeter grip strength in CIDP: a responsive tool that rapidly measures the effect of IVIG – the ICE study. *European Journal of Neurology*. 2013;20(5):748-755. doi:10.1111/j.1468-1331.2012.03851.x

Supplementary Table 1: Smartphone Real-World Cohort Characteristics

		Missing / n	Overall
**n (%)**		0 / 20	20 (100)
**Sex, n (%)**	**w**	0 / 20	7 (35)
	**m**		13 (65)
**Age at inclusion [y], median [Q1, Q3]**		0 / 20	61 [53, 74.3]
**Baseline INCAT Disability Score, median [Q1, Q3]**		0 / 20	2.5 [1.8, 3.3]
**Baseline I-RODS, median [Q1, Q3]**		0 / 20	32 [26.8, 41.5]
**Baseline MRC-sumscore, median [Q1, Q3]**		0 / 20	58 [56, 60]
**Immunoglobulin treatment interval [d], median [Q1, Q3]**		0 / 20	28 [28, 42]
**Average steps per day per subject, mean (SD)**		0 / 20	4546 (3380)
**Maximum steps per day per subject, mean (SD)**		0 / 20	13442 (7981)
**WHOQOL-BREF completed, n (%)**		0 / 20	17 (85)

n = quantity, w = women, m = men, y = year, d = days, SD = standard deviation, Q1 = lower quartile, Q3 = upper quartile, MRC= Medical Research Council, I-RODS = Inflammatory Rasch-built Overall Disability Scale, INCAT = Inflammatory Neuropathy Cause and Treatment, WHOQOL-BREF = World Health Organization Quality of Life-BREF

Supplementary Table 2: Adherence Data

Sequential ID	Hours Total	Hours With Data	% of Hours With Data	Number of Days	Number of Days Without Data	Days Adherence Criteria Met	% of Days Adherence Criteria Met
1	4009	3927	98.0	168	0	164	97.6
2	4111	4008	97.5	172	0	165	95.9
3	4060	3569	87.9	170	5	149	87.6
4	3934	3896	99.0	165	1	161	97.6
5	3602	2700	75.0	151	7	115	76.2
6	5479	3517	64.2	229	34	122	53.3
7	5040	5002	99.2	211	0	210	99.5
8	4030	4005	99.4	169	0	167	98.8
9	4208	4161	98.9	176	0	175	99.4
10	4197	4185	99.7	176	1	175	99.4
11	5271	5184	98.3	220	0	215	97.7
12	4190	4108	98.0	175	0	173	98.9
13	4023	3988	99.1	168	0	167	99.4
14	4686	4628	98.8	196	0	195	99.5
15	3927	3700	94.2	164	0	147	89.6
16	4215	3145	74.6	176	41	132	75
17	5002	4947	98.9	209	0	209	100
18	4032	2719	67.4	169	6	129	76.3
19	4048	4022	99.4	169	0	168	99.4
20	5832	5639	96.7	244	3	235	96.3
21	3969	3884	97.9	166	0	164	98.8
22	4680	4572	97.7	196	0	189	96.4
23	4336	2397	55.3	181	68	97	53.6
24	6519	6461	99.1	272	1	270	99.3
25	3532	3471	98.3	148	0	143	96.6
26	3920	3889	99.2	164	0	163	99.4
27	4978	4521	90.8	208	11	188	90.4
28	4024	3986	99.1	168	0	164	97.6
29	3951	3938	99.7	165	0	165	100
30	4179	4050	96.9	175	0	168	96
31	4065	3919	96.4	170	0	163	95.9
32	4096	3868	94.4	171	5	160	93.6
33	4032	3946	97.9	169	0	162	95.9
34	4528	3780	83.5	189	29	158	83.6
35	4226	4136	97.9	177	0	170	96
36	5340	5167	96.8	223	0	213	95.5
37	4835	4313	89.2	202	17	180	89.1
38	4033	3980	98.7	169	1	166	98.2
39	4020	3954	98.4	168	0	164	97.6
40	4513	4447	98.5	189	0	185	97.9
41	4680	4655	99.5	196	0	195	99.5
42	5351	4414	82.5	224	2	170	75.9
43	4713	4670	99.1	197	0	197	100
44	4870	4849	99.6	204	1	202	99
45	3992	3935	98.6	167	0	164	98.2

Supplementary Table 3: Post-hoc Comparison Results

Variable 1	Variable 2	Coefficient	Coefficient CI Lower	Coefficient CI Upper	P-Value	Adjusted P-Value	Correlation Method	Significance
		Smartwatch Parameters (Holm Correction)						
Mean Steps	Median Steps	0.99	0.98	0.99	P < 0.001	P < 0.001	Pearson	****
Mean Steps	Maximum Steps	0.85	0.76	0.92	P < 0.001	P < 0.001	Pearson	****
Mean Steps	Mean Moderate Activity	0.83	0.66	0.92	P < 0.001	P < 0.001	Spearman	****
Mean Steps	Median Moderate Activity	0.83	0.7	0.91	P < 0.001	P < 0.001	Spearman	****
Mean Steps	Maximum Moderate Activity	0.64	0.41	0.8	P < 0.001	P < 0.001	Spearman	****
Median Steps	Maximum Steps	0.78	0.65	0.87	P < 0.001	P < 0.001	Pearson	****
Median Steps	Mean Moderate Activity	0.81	0.63	0.92	P < 0.001	P < 0.001	Spearman	****
Median Steps	Median Moderate Activity	0.84	0.72	0.92	P < 0.001	P < 0.001	Spearman	****
Median Steps	Maximum Moderate Activity	0.58	0.33	0.78	P < 0.001	P < 0.001	Spearman	***
Maximum Steps	Mean Moderate Activity	0.73	0.53	0.86	P < 0.001	P < 0.001	Spearman	****
Maximum Steps	Median Moderate Activity	0.69	0.48	0.81	P < 0.001	P < 0.001	Spearman	****
Maximum Steps	Maximum Moderate Activity	0.7	0.49	0.85	P < 0.001	P < 0.001	Spearman	****
Mean Moderate Activity	Median Moderate Activity	0.93	0.84	0.97	P < 0.001	P < 0.001	Spearman	****
Mean Moderate Activity	Maximum Moderate Activity	0.8	0.66	0.88	P < 0.001	P < 0.001	Spearman	****
Median Moderate Activity	Maximum Moderate Activity	0.63	0.39	0.78	P < 0.001	P < 0.001	Spearman	****
		Clinical vs Smartwatch (Benjamini-Hochberg Correction)						
Mean Steps	MRC Score	0.27	-0.05	0.54	0.077	0.089	Spearman	
Mean Steps	I-RODS Score	0.65	0.4	0.81	P < 0.001	P < 0.001	Spearman	****
Mean Steps	INCAT Score	-0.43	-0.68	-0.09	0.004	0.014	Spearman	*
Mean Steps	Dominant Grip Strength	0.26	-0.03	0.51	0.09	0.1	Spearman	
Mean Steps	Non-Dominant Grip Strength	0.33	0.01	0.57	0.031	0.044	Spearman	*
Median Steps	MRC Score	0.25	-0.04	0.51	0.1	0.107	Spearman	

Median Steps	I-RODS Score	0.59	0.35	0.75	P < 0.001	P < 0.001	Spearman	***
Median Steps	INCAT Score	-0.38	-0.62	-0.09	0.011	0.021	Spearman	*
Median Steps	Dominant Grip Strength	0.22	-0.03	0.45	0.152	0.152	Spearman	
Median Steps	Non-Dominant Grip Strength	0.29	0	0.52	0.057	0.068	Spearman	
Maximum Steps	MRC Score	0.37	0.04	0.63	0.014	0.024	Spearman	*
Maximum Steps	I-RODS Score	0.74	0.53	0.89	P < 0.001	P < 0.001	Spearman	****
Maximum Steps	INCAT Score	-0.54	-0.75	-0.25	P < 0.001	P < 0.001	Spearman	***
Maximum Steps	Dominant Grip Strength	0.41	0.11	0.67	0.006	0.015	Spearman	*
Maximum Steps	Non-Dominant Grip Strength	0.45	0.13	0.67	0.002	0.009	Spearman	**
Mean Moderate Activity	MRC Score	0.31	0.03	0.55	0.041	0.056	Spearman	
Mean Moderate Activity	I-RODS Score	0.61	0.36	0.78	P < 0.001	P < 0.001	Spearman	***
Mean Moderate Activity	INCAT Score	-0.4	-0.64	-0.08	0.007	0.016	Spearman	*
Mean Moderate Activity	Dominant Grip Strength	0.3	0.05	0.52	0.049	0.062	Spearman	
Mean Moderate Activity	Non-Dominant Grip Strength	0.41	0.16	0.62	0.006	0.015	Spearman	*
Median Moderate Activity	MRC Score	0.24	-0.07	0.5	0.127	0.131	Spearman	
Median Moderate Activity	I-RODS Score	0.56	0.29	0.76	P < 0.001	P < 0.001	Spearman	***
Median Moderate Activity	INCAT Score	-0.39	-0.65	-0.08	0.01	0.021	Spearman	*
Median Moderate Activity	Dominant Grip Strength	0.31	0.03	0.54	0.045	0.059	Spearman	
Median Moderate Activity	Non-Dominant Grip Strength	0.41	0.13	0.62	0.006	0.015	Spearman	*
Maximum Moderate Activity	MRC Score	0.34	0.02	0.59	0.025	0.037	Spearman	*
Maximum Moderate Activity	I-RODS Score	0.57	0.31	0.76	P < 0.001	P < 0.001	Spearman	***
Maximum Moderate Activity	INCAT Score	-0.35	-0.59	-0.02	0.023	0.036	Spearman	*
Maximum Moderate Activity	Dominant Grip Strength	0.37	0.07	0.61	0.016	0.026	Spearman	*
Maximum Moderate Activity	Non-Dominant Grip Strength	0.41	0.12	0.65	0.006	0.015	Spearman	*

Smartwatch & Clinical vs QoL (Benjamini-Hochberg Correction)

Mean Steps	WHOQOL Physical Health	0.49	0.23	0.68	P < 0.001	0.003	Spearman	**
Mean Steps	WHOQOL Psychological	0.3	-0.03	0.56	0.052	0.062	Spearman	
Mean Steps	WHOQOL Social Relationships	0.35	0.02	0.59	0.021	0.038	Spearman	*
Mean Steps	WHOQOL Environment	0.34	0.04	0.59	0.026	0.038	Spearman	*

Maximum Steps	WHOQOL Physical Health	0.55	0.3	0.74	P < 0.001	0.002	Spearman	**
Maximum Steps	WHOQOL Psychological	0.38	0.1	0.61	0.011	0.026	Spearman	*
Maximum Steps	WHOQOL Social Relationships	0.35	0.06	0.59	0.023	0.038	Spearman	*
Maximum Steps	WHOQOL Environment	0.42	0.14	0.66	0.005	0.013	Spearman	*
Mean Moderate Activity	WHOQOL Physical Health	0.58	0.35	0.74	P < 0.001	P < 0.001	Spearman	***
Mean Moderate Activity	WHOQOL Psychological	0.32	0.01	0.57	0.034	0.045	Spearman	*
Mean Moderate Activity	WHOQOL Social Relationships	0.33	0.04	0.58	0.029	0.04	Spearman	*
Mean Moderate Activity	WHOQOL Environment	0.4	0.13	0.64	0.007	0.018	Spearman	*
Maximum Moderate Activity	WHOQOL Physical Health	0.54	0.27	0.73	P < 0.001	0.002	Spearman	**
Maximum Moderate Activity	WHOQOL Psychological	0.32	-0.01	0.62	0.035	0.045	Spearman	*
Maximum Moderate Activity	WHOQOL Social Relationships	0.34	0.07	0.56	0.025	0.038	Spearman	*
Maximum Moderate Activity	WHOQOL Environment	0.35	0	0.61	0.023	0.038	Spearman	*
Dominant Grip Strength	WHOQOL Physical Health	0.29	-0.05	0.59	0.064	0.073	Spearman	
Dominant Grip Strength	WHOQOL Psychological	0.34	0.02	0.62	0.026	0.038	Spearman	*
Dominant Grip Strength	WHOQOL Social Relationships	0.17	-0.13	0.46	0.286	0.286	Spearman	
Dominant Grip Strength	WHOQOL Environment	0.24	-0.04	0.49	0.127	0.136	Spearman	
MRC Score	WHOQOL Physical Health	0.42	0.1	0.67	0.005	0.013	Spearman	*
MRC Score	WHOQOL Psychological	0.25	-0.06	0.49	0.112	0.124	Spearman	
MRC Score	WHOQOL Social Relationships	0.22	-0.07	0.5	0.158	0.164	Spearman	
MRC Score	WHOQOL Environment	0.5	0.24	0.7	P < 0.001	0.003	Spearman	**
I-RODS Score	WHOQOL Physical Health	0.7	0.47	0.82	P < 0.001	P < 0.001	Spearman	****
I-RODS Score	WHOQOL Psychological	0.52	0.26	0.73	P < 0.001	0.002	Spearman	**
I-RODS Score	WHOQOL Social Relationships	0.43	0.14	0.67	0.004	0.012	Spearman	*
I-RODS Score	WHOQOL Environment	0.52	0.21	0.71	P < 0.001	0.002	Spearman	**
INCAT Score	WHOQOL Physical Health	-0.51	-0.7	-0.27	P < 0.001	0.002	Spearman	**
INCAT Score	WHOQOL Psychological	-0.37	-0.6	-0.07	0.014	0.03	Spearman	*
INCAT Score	WHOQOL Social Relationships	-0.32	-0.57	-0.07	0.037	0.045	Spearman	*
INCAT Score	WHOQOL Environment	-0.35	-0.58	-0.07	0.023	0.038	Spearman	*

Supplementary Figure 1: Cohort overview of steps and moderate activity aggregation in the analyzed trial population (n=43)

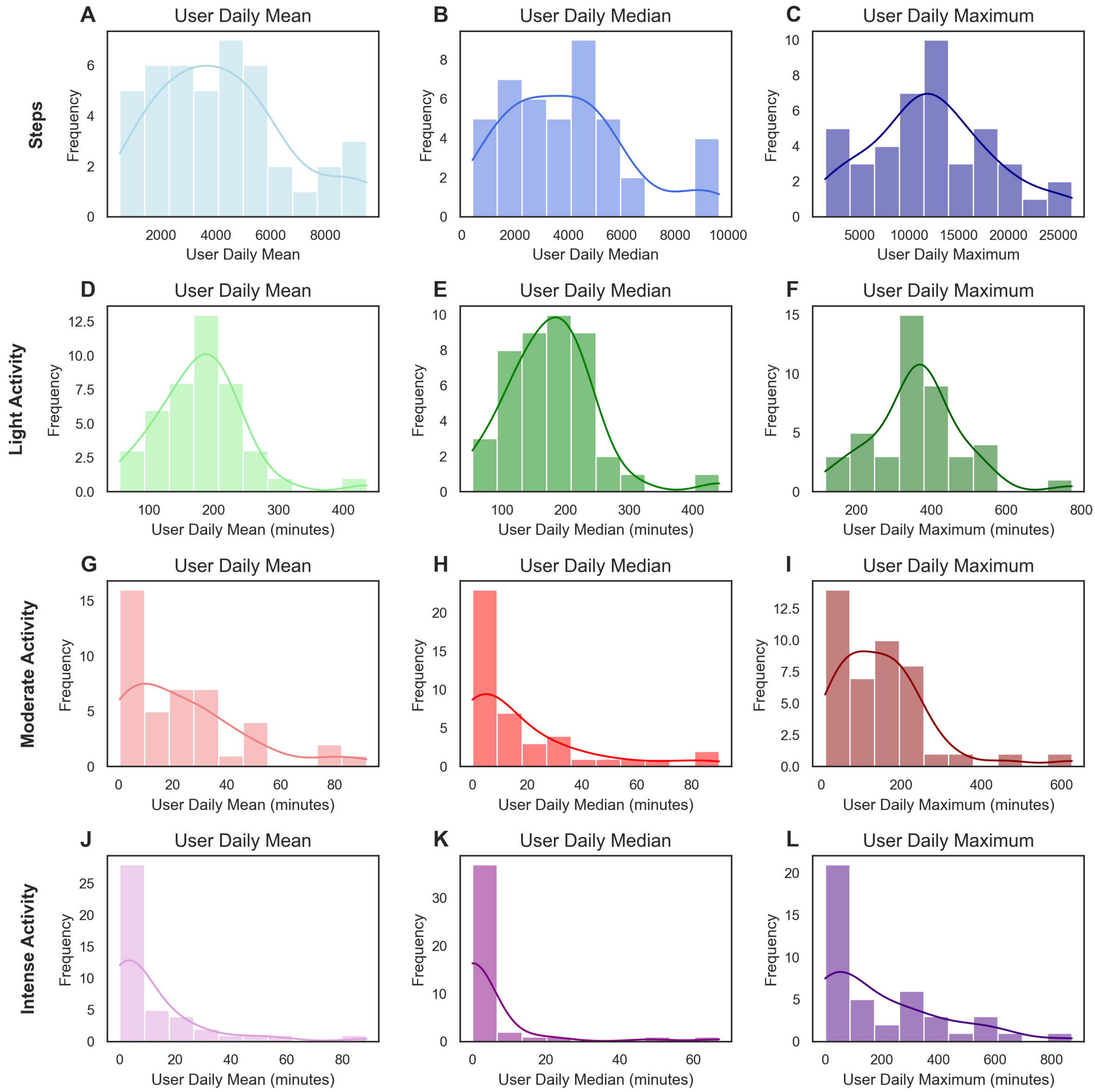

Supplementary Figure 1: Cohort overview of steps and moderate activity aggregation in the analyzed trial population (n=43).

This figure presents histograms with KDE illustrating the distribution of various step and moderate activity metrics in the EMDA-CIDP population (n=43).

(A-C) show the distributions of mean steps, median steps, and maximum steps per user, respectively. These histograms provide an overview of how step metrics are distributed across the study population, highlighting the variability and central tendency within the cohort.

(D-F) illustrate mean light activity, median light activity, and maximum light activity per user, respectively. Almost all patients reach this activity level frequently with a central tendency.

(G-I) depict the distributions of mean moderate activity, median moderate activity, and maximum moderate activity per user, respectively. All distributions show a left skew with most users on the lower end of the distribution in terms of activity time.

(J-L) show mean intense activity, median intense activity, and maximum intense activity per user, respectively. These plots illustrate the distribution of high-intensity activities, which are generally lower across the population and seem not to occur on a daily basis.

CIDP: Chronic Inflammatory Demyelinating Polyradiculoneuropathy, KDE: Kernel Density Estimate

Supplementary Figure 2: Smartwatch adherence among study participants

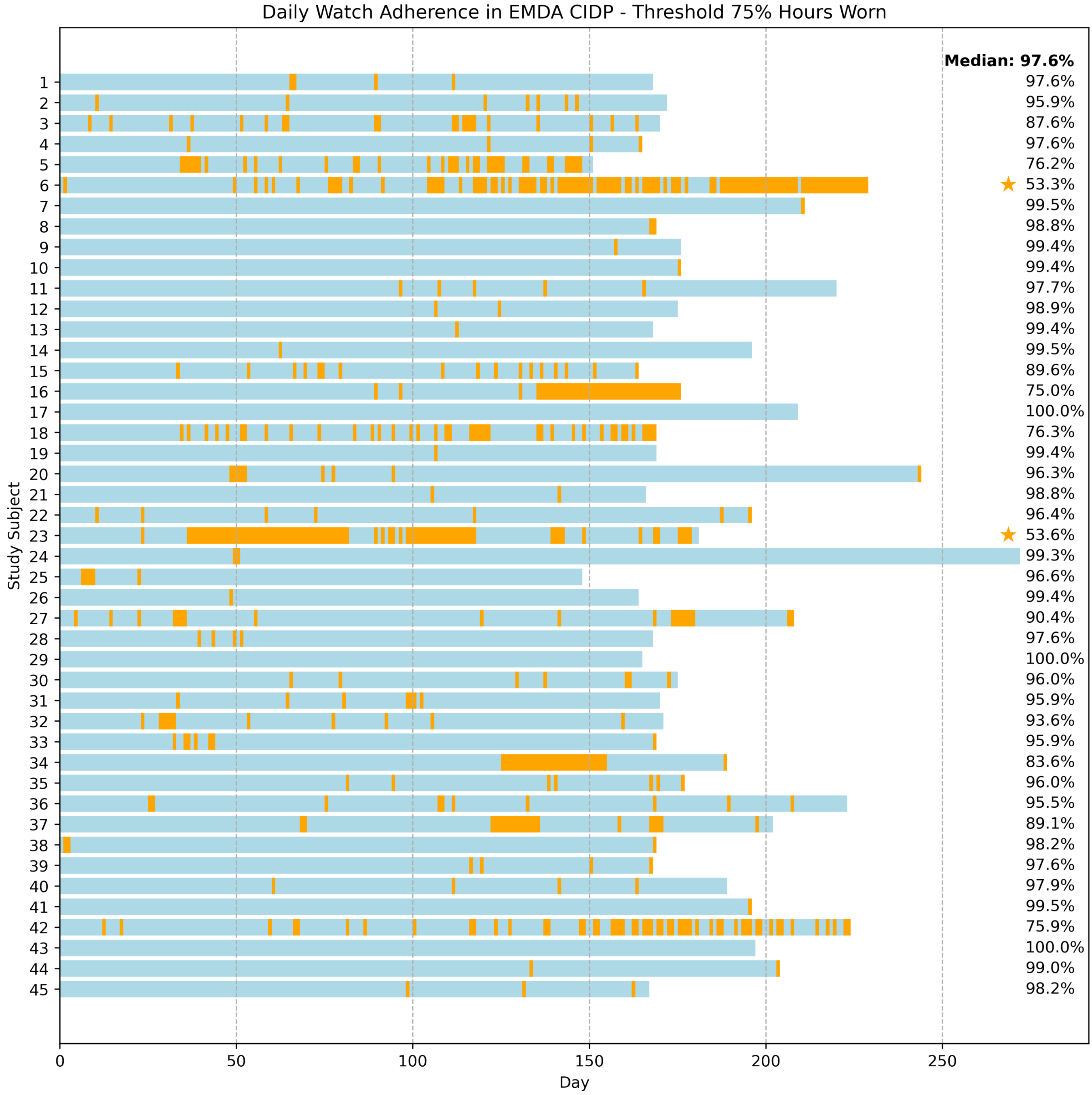

Supplementary Figure 2: Smartwatch adherence among study participants.

This figure illustrates the daily adherence of 45 patients to the smartwatch wear-time criteria during the study. The criteria required a minimum of 75% wear-time during wake hours (6 am - 11 pm). Days where the criteria were met are marked in blue, while days not meeting the criteria are shown in orange. Two patients who had less than 75% of days meeting the criteria (indicated by orange stars) were excluded from further analysis. The percentage of days meeting adherence criteria is written next to each patient’s respective bar at the right with median adherence across all patients at 97.6%. Daily and hourly adherence data is also available in a detailed tabular view in Supplementary Table 2.

Both participants failed to meet this target because they stopped wearing the device at night and wore it more irregularly thereafter, one of the patients additionally reported syncing problems.

Supplementary Figure 3: Screening matrix of spearman correlation coefficients between smartwatch activity and clinical scores

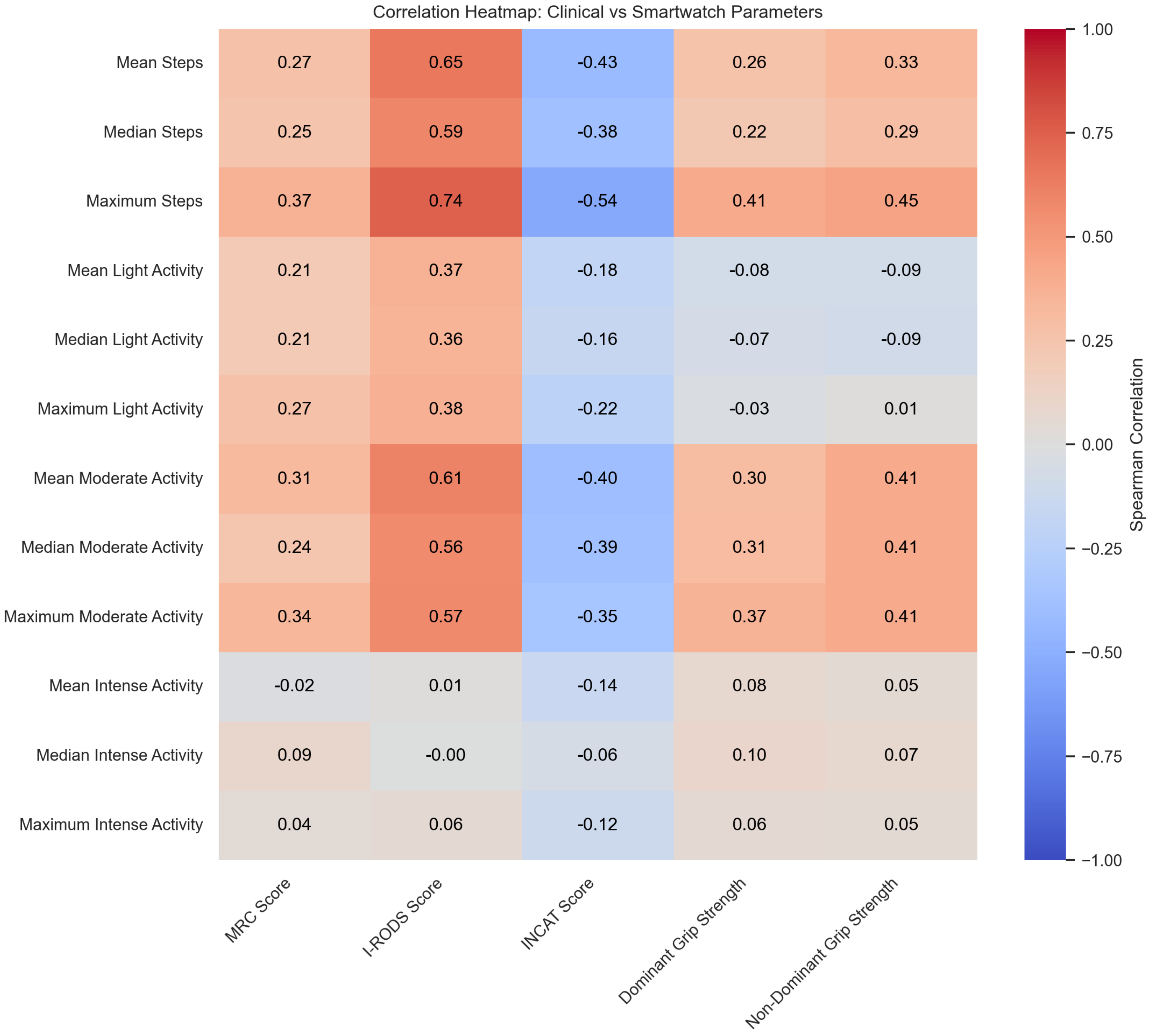

Supplementary Figure 3: Screening matrix of spearman correlation coefficients between smartwatch activity and clinical scores.

This heatmap displays Spearman correlation coefficients between various smartwatch activity metrics and clinical scores in the EMDA-CIDP population (n=43). Maximum steps and moderate activity showed the strongest correlations, particularly with the I-RODS Score, indicating these metrics may be useful for assessing CIDP severity and were chosen for further exploration.

CIDP: Chronic Inflammatory Demyelinating Polyradiculoneuropathy, INCAT: Inflammatory Neuropathy Cause and Treatment, I-RODS: Inflammatory Rasch-built Overall Disability Scale, MRC: Medical Research Council.

Supplementary Figure 4: Correlation heatmap for hypothesis generation on smartwatch activity, clinical parameters, and quality of life in CIDP patients.

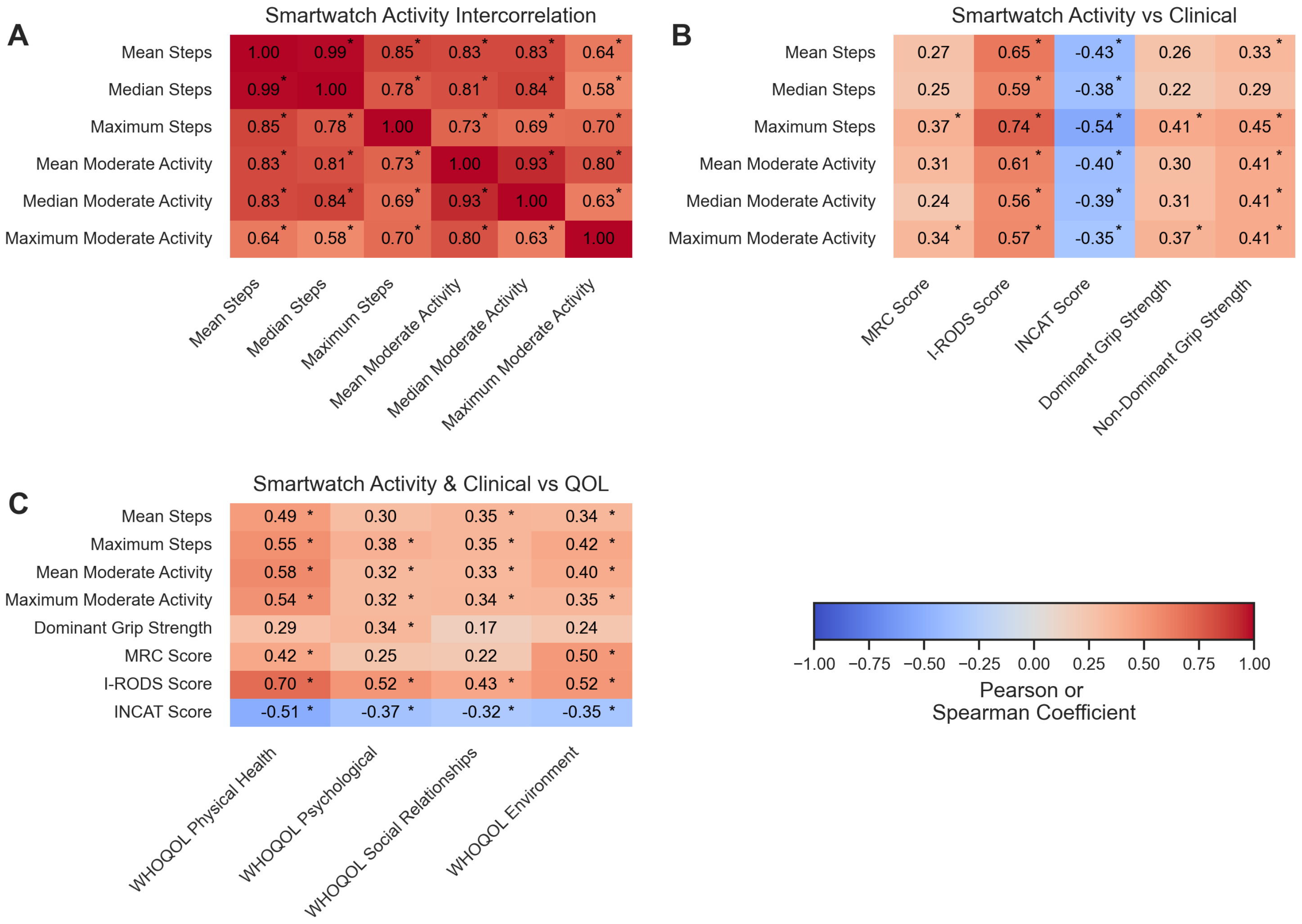

Supplementary Figure 4: Correlation heatmap for hypothesis generation on smartwatch activity, clinical parameters, and quality of life in CIDP patients.

(A) Heatmap displaying the intercorrelation between various aggregated smartwatch metrics, including mean, median, and maximum steps, as well as mean, median, and maximum moderate activity. Strong positive correlations are observed, particularly among step and moderate activity measures.

(B) Heatmap illustrating the correlation between smartwatch activity metrics and clinical parameters such as MRC-sumscore, I-RODS and INCAT Score, and grip strength. Maximum steps and activity metrics show significant correlations with all tested clinical measures.

(C) Heatmap showing the correlation between selected smartwatch activity and clinical metrics, compared with quality of life domains from the WHOQOL-BREF. Significant correlations are observed across multiple QOL domains, with some smartwatch and clinical measures showing consistent correlations with all domains.

Red indicates positive correlations, while blue indicates negative correlations. Stars denote statistically significant correlations after applying either the more conservative Holm correction (A) or the FDR Benjamini-Hochberg correction for hypothesis generation (B and C) across multiple comparisons. Pearson or Spearman correlation coefficients were used based on data distribution. All unadjusted and adjusted p-values as well as confidence interval estimates can also be found alongside the correlation and correction method used for each pair in Supplementary Tab. 2. Supplementary Figures 5 and 6 provide confidence interval estimates for some of these correlations.

CIDP: Chronic Inflammatory Demyelinating Polyradiculoneuropathy, FDR: False Discovery Rate, I-RODS: Inflammatory Rasch-built Overall Disability Scale, INCAT: Inflammatory Neuropathy Cause and Treatment, MRC: Medical Research Council, QOL: Quality of Life, WHOQOL-BREF: World Health Organization Quality of Life-BREF.

Supplementary Figure 5: Bootstrapped spearman correlation coefficients of smartwatch parameters with clinical scores

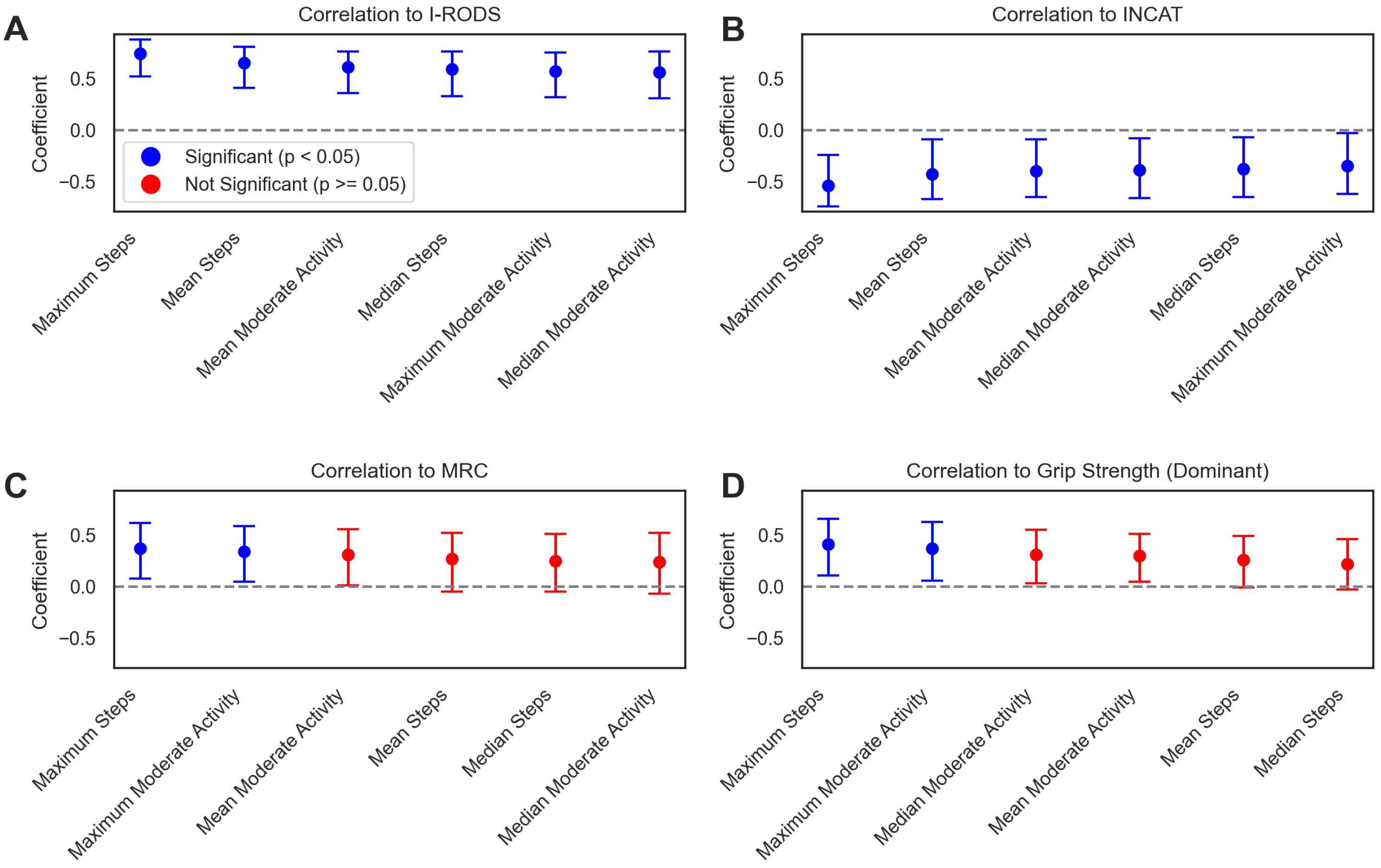

Supplementary Figure 5: Bootstrapped spearman correlation coefficients of smartwatch parameters with clinical scores

This figure shows bootstrapped Spearman correlation coefficients between various smartwatch activity parameters and clinical scores, with confidence intervals. Panels (A-D) represent correlations with I-RODS, INCAT, MRC, and dominant hand grip strength, respectively. The confidence intervals generally overlap, highlighting the limited ability to definitively identify the best metric.

However, maximum steps consistently appeared the most promising, being the only metric to show significant correlations across all clinical scores. All p-values were FDR Benjamini-Hochberg corrected. Detailed results of the tests and preadjusted as well as adjusted p-values are available in Supplementary Table 3.

FDR: False Discovery Rate, INCAT: Inflammatory Neuropathy Cause and Treatment, I-RODS: Inflammatory Rasch-built Overall Disability Scale, MRC: Medical Research Council.

Supplementary Figure 6: Bootstrapped spearman correlation coefficients of selected clinical and smartwatch variables with WHOQOL domains

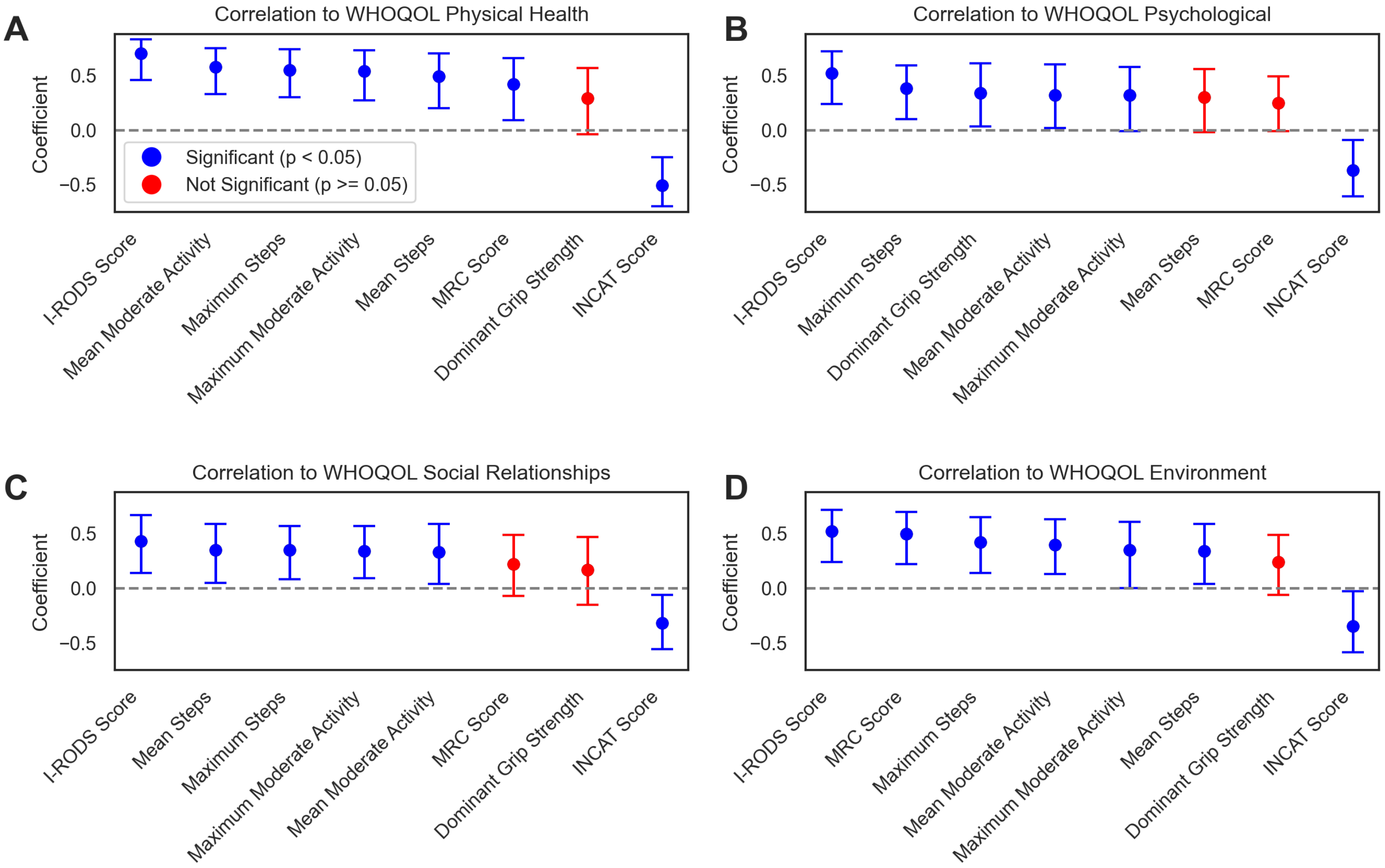

Supplementary Figure 6: Bootstrapped spearman correlation coefficients of selected clinical and smartwatch variables with WHOQOL domains

This figure shows bootstrapped Spearman correlation coefficients between various smartwatch activity parameters and WHOQOL domains, with confidence intervals. Panels (A-D) represent correlations with WHOQOL Physical Health, Psychological, Social Relationships, and Environment domains, respectively. The confidence intervals generally overlap, highlighting the limited ability to compare metrics.

All p-values were FDR Benjamini-Hochberg corrected. Detailed results of the tests and preadjusted as well as adjusted p values are available in Supplementary Table 3.

FDR: False Discovery Rate, INCAT: Inflammatory Neuropathy Cause and Treatment, I-RODS: Inflammatory Rasch-built Overall Disability Scale, MRC: Medical Research Council, WHOQOL: World Health Organization Quality of Life.

Supplementary Figure 7: Heatmap of spearman correlations between percentiles of steps, clinical/QOL values, age, and BMI in CIDP patients

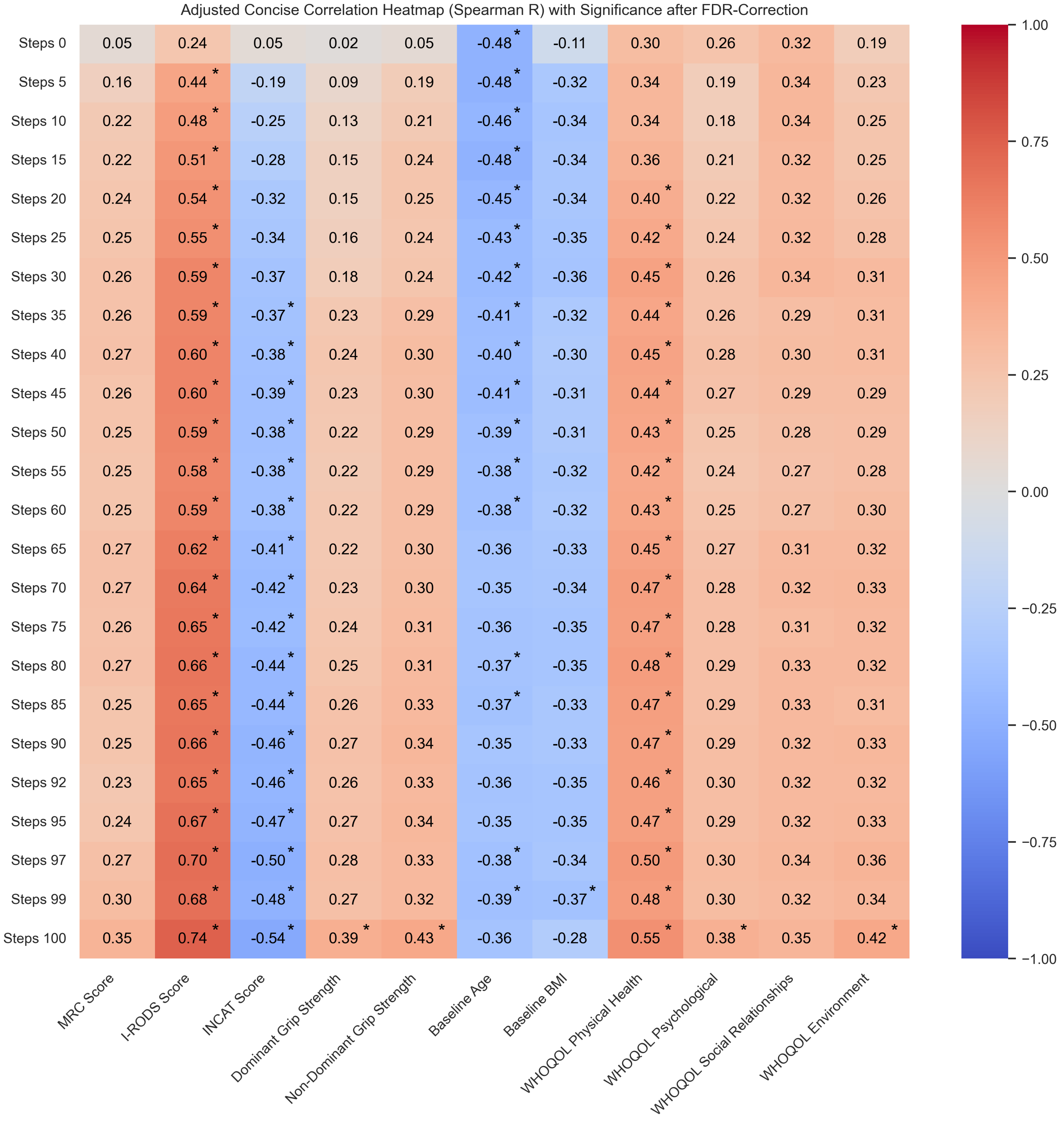

Supplementary Figure 7: Heatmap of spearman correlations between percentiles of steps, clinical/QOL values, age, and BMI in CIDP patients

This heatmap displays Spearman correlation coefficients between various percentiles of daily steps in CIDP patients and clinical as well as QOL measures. Significant correlations after FDR Benjamini-Hochberg correction are indicated with stars. The analysis reveals how different levels of daily steps correlate with clinical scores, QOL, age, and BMI, offering insights into the potential impact of physical activity on patient well-being and how it interacts with demographic factors.

BMI: Body Mass Index, CIDP: Chronic Inflammatory Demyelinating Polyradiculoneuropathy, FDR: False Discovery Rate, INCAT: Inflammatory Neuropathy Cause and Treatment, I-RODS: Inflammatory Rasch-built Overall Disability Scale, MRC: Medical Research Council, WHOQOL: World Health Organization Quality of Life.

Supplementary Figure 8: Absolute spearman correlations of moderate activity percentiles with clinical scores and QOL results

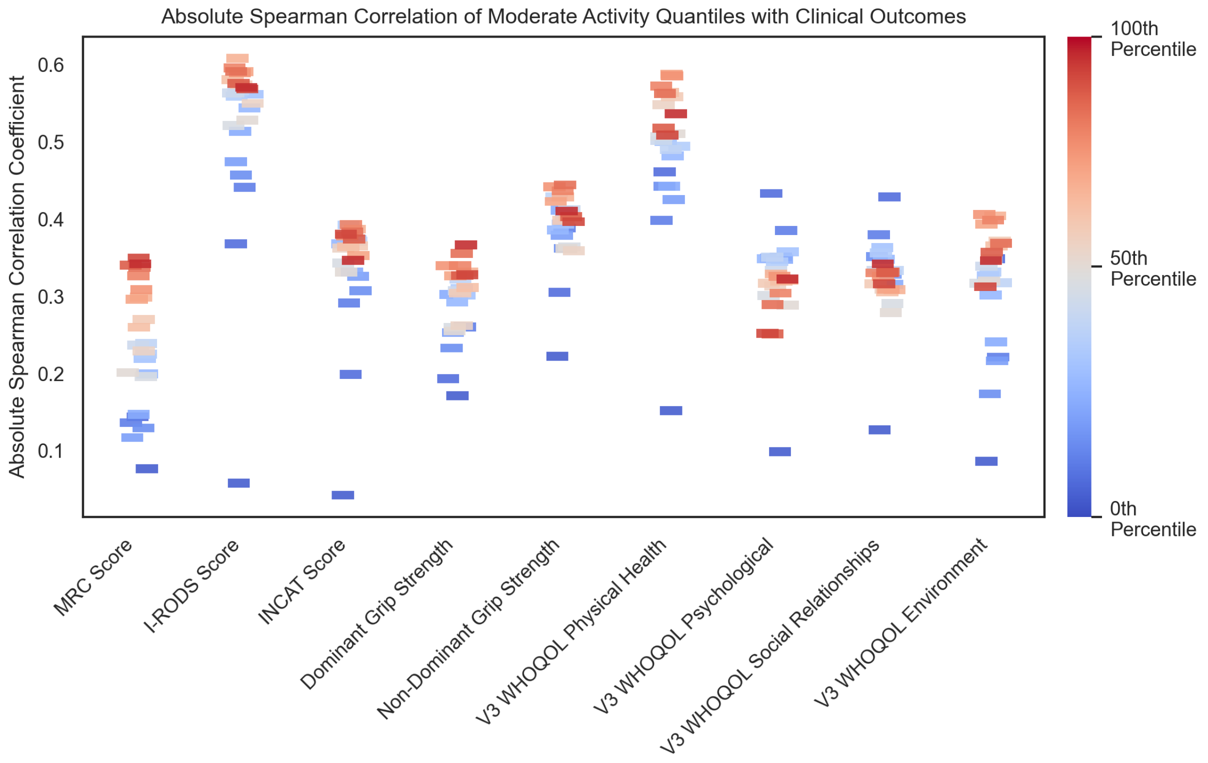

Supplementary Figure 8: Absolute spearman correlations of moderate activity percentiles with clinical scores and QOL results

The strip plot presents the absolute (non-negative) Spearman correlations of different moderate activity percentiles (in increments of ten), including maximum moderate activity (darkest red) and other percentiles, with various clinical CIDP parameters (e.g., I-RODS or INCAT Score) and QOL domains (e.g., WHOQOL Physical Health). The correlation values are represented as rectangles on the plot, with higher percentiles indicated in red and lower percentiles in blue. Supplementary Figure 9 provides a detailed heatmap of these correlations, including statistical significances. Generally, maximum moderate activity appears to have comparably high correlations across several outcomes.

CIDP: Chronic Inflammatory Demyelinating Polyradiculoneuropathy, INCAT: Inflammatory Neuropathy Cause and Treatment, I-RODS: Inflammatory Rasch-built Overall Disability Scale, MRC: Medical Research Council, WHOQOL: World Health Organization Quality of Life.

Supplementary Figure 9: Heatmap of spearman correlations between percentiles of moderate activity, clinical/QOL values, age, and BMI in CIDP patients

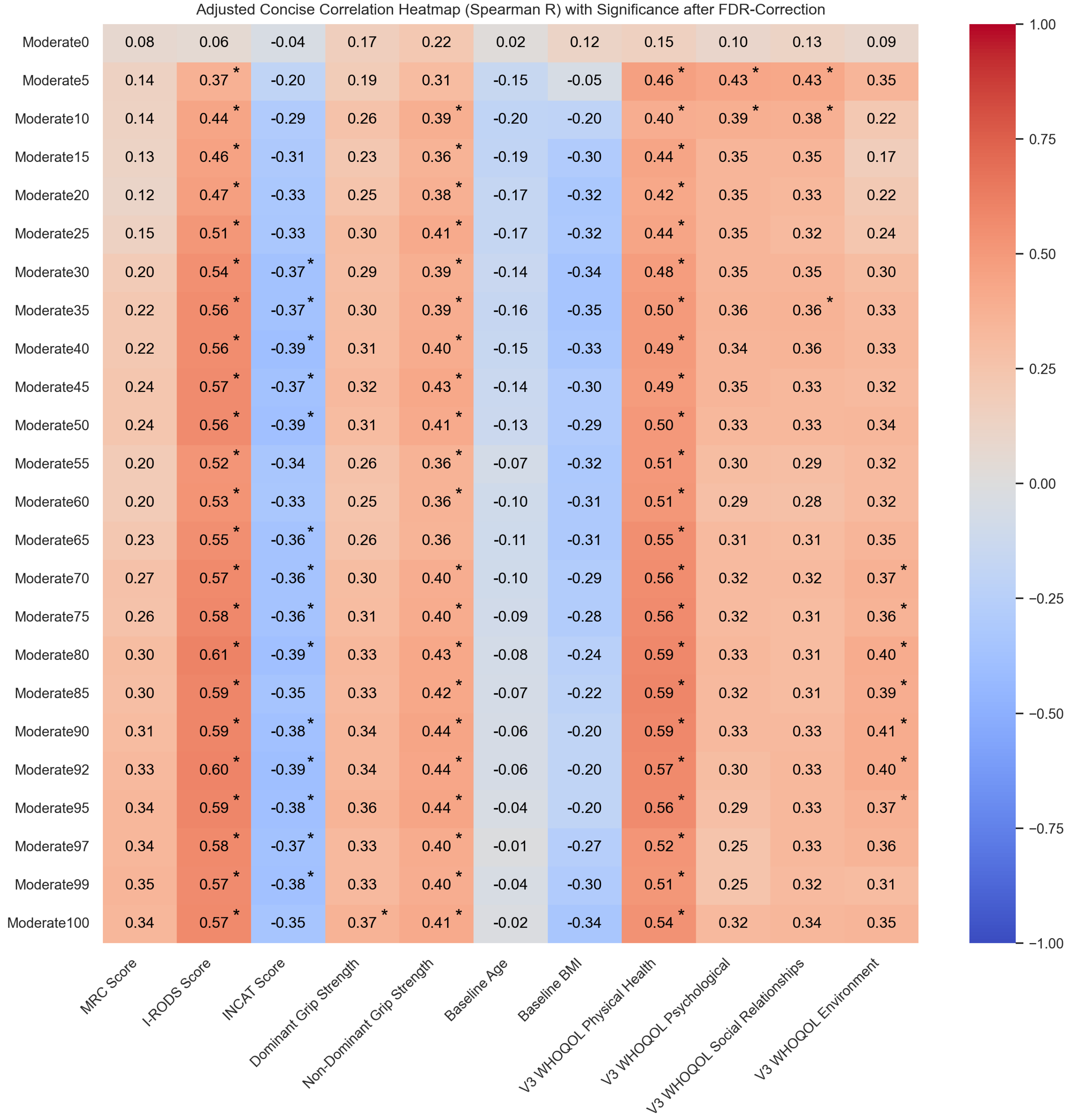

Supplementary Figure 9: Heatmap of spearman correlations between percentiles of moderate activity, clinical/QOL values, age, and BMI in CIDP patients

This heatmap displays Spearman correlation coefficients between various percentiles of daily moderate activity in CIDP patients and clinical measures, Quality of Life (QOL) domains, age, and Body Mass Index (BMI). Significant correlations after FDR Benjamini-Hochberg correction are indicated with stars.

BMI: Body Mass Index, CIDP: Chronic Inflammatory Demyelinating Polyradiculoneuropathy, FDR: False Discovery Rate, INCAT: Inflammatory Neuropathy Cause and Treatment, I-RODS: Inflammatory Rasch-built Overall Disability Scale, MRC: Medical Research Council, WHOQOL: World Health Organization Quality of Life.

Supplementary Figure 10: Ridge regression coefficients for I-RODS prediction in CIDP patients

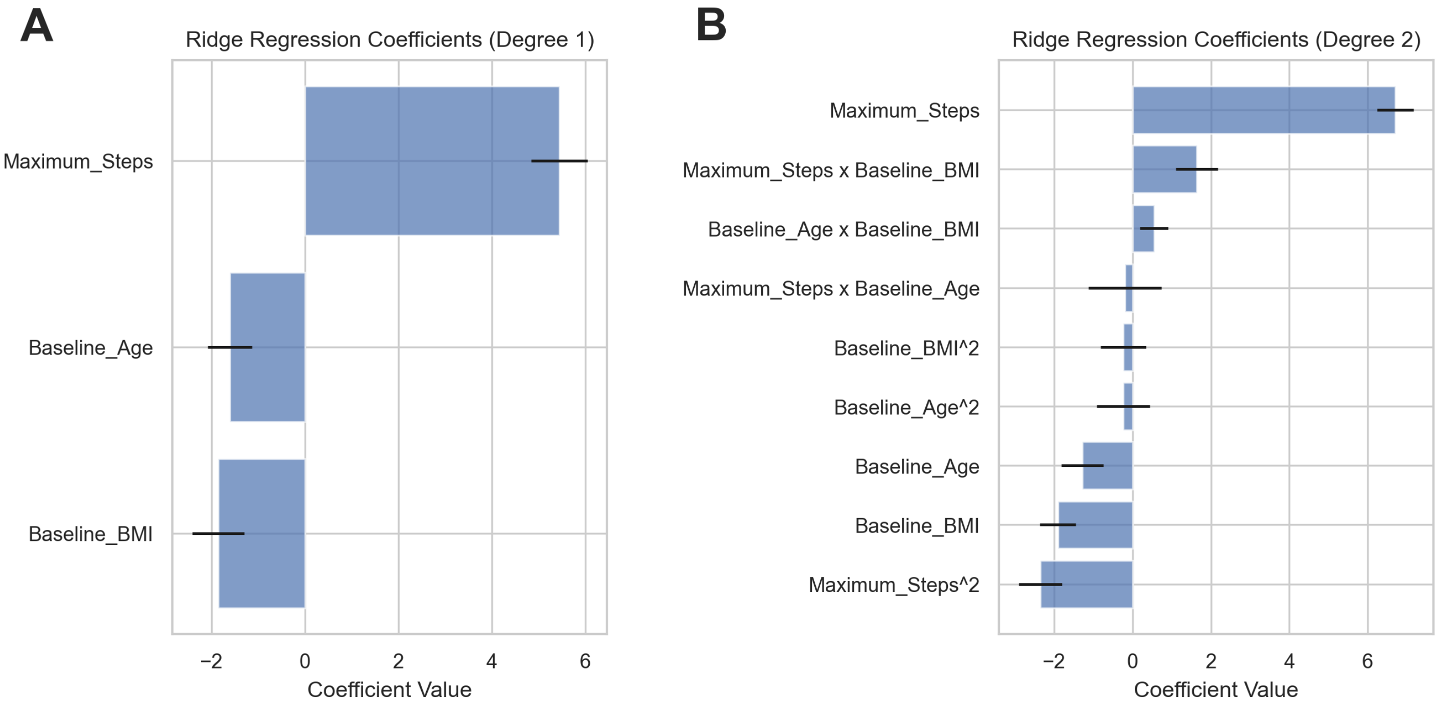

Supplementary Figure 10: Ridge regression coefficients for I-RODS prediction in CIDP patients

This figure presents the results of a 5-fold cross-validated Ridge Regression analysis to predict I-RODS scores in patients with CIDP, using polynomial features of degree 1 and 2. Coefficients are shown with their standard deviations across the folds.

(A) In the first-degree polynomial Ridge Regression model, maximum daily steps is positively correlated with I-RODS, while potential confounders such as baseline age and baseline BMI show a small negative influence.

(B) The second-degree polynomial Ridge Regression model highlights the negative effect of the squared Maximum Steps term, which aligns with the observed leveling off effect of increased steps on I-RODS scores. This suggests that after a certain point, additional steps contribute less positively or may even have a diminishing return in terms of I-RODS improvement. Minimal significant interactions between other variables were observed, indicating that the second-degree model offers only marginal additional insight over the first-degree model.

Model performance:

- **Degree 1**: Average MSE: 50.06 ± 18.71, Average R^2: 0.41 ± 0.12
- **Degree 2**: Average MSE: 44.58 ± 16.19, Average R^2: 0.46 ± 0.18

BMI: Body Mass Index, CIDP: Chronic Inflammatory Demyelinating Polyradiculoneuropathy, I-RODS: Inflammatory Rasch-built Overall Disability Scale

Supplementary Figure 11: Scatter plots of maximum daily steps correlated with clinical scores in CIDP patients and a real-world cohort using LOESS regression

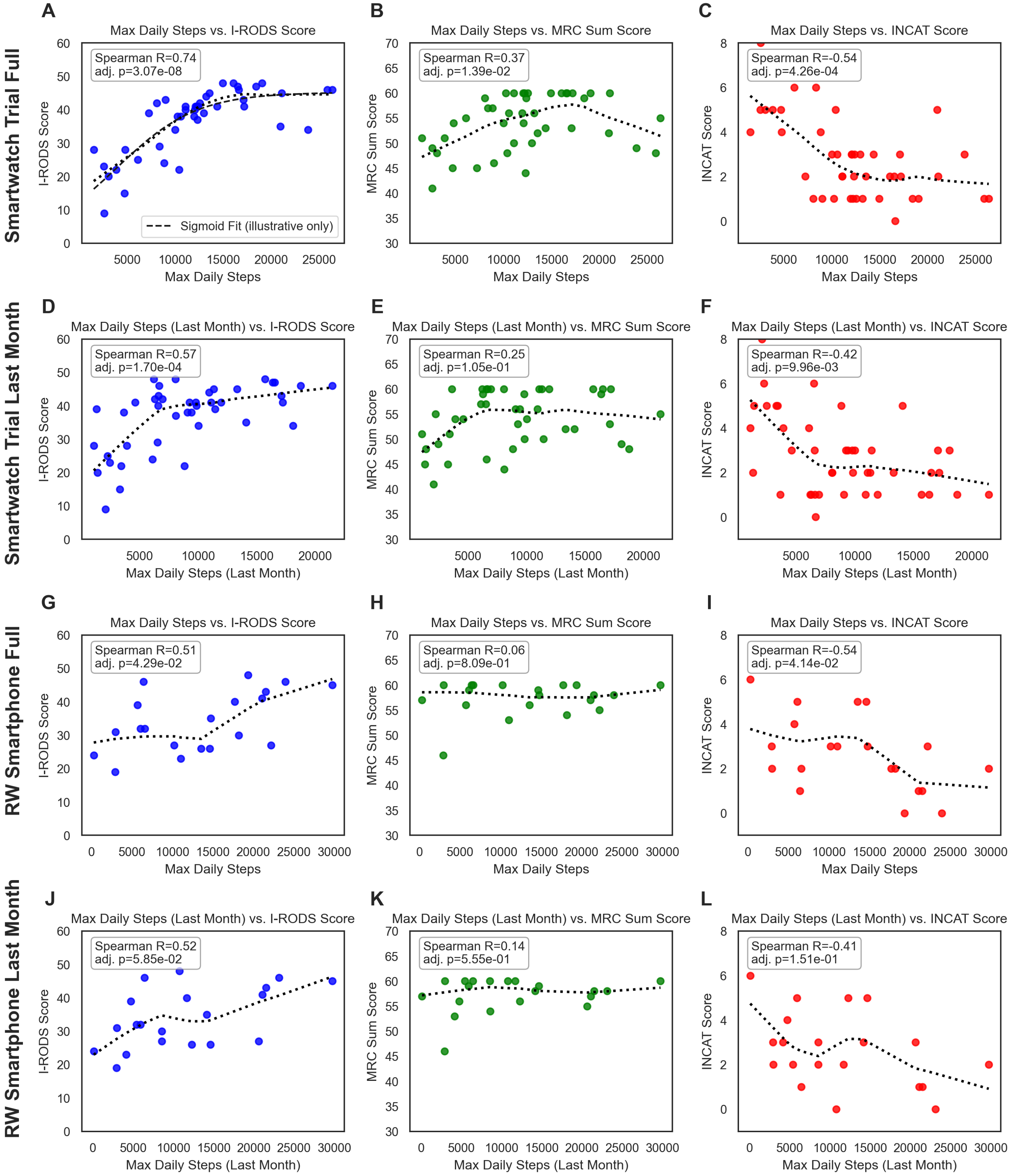

See next page for legend.

Supplementary Figure 11: Scatter plots of maximum daily steps correlated with clinical scores in CIDP patients and a real-world cohort using LOESS regression

This figure presents scatter plots illustrating the relationship between maximum daily steps and three clinical scores: I-RODS (blue – left column), MRC-sumscore (green – middle column), and INCAT Score (red – right column). The analysis is conducted across different observation periods and cohorts. All reported R values in the graphs are row-wise Holm-adjusted Spearman correlation coefficients, reflecting the rank-based relationship between maximum steps and the clinical scores. The LOESS regression trendlines in these plots provide a more technically accurate representation of the relationships, capturing potential non-linear trends that linear models may not adequately reflect, especially given the ordinal nature of the MRC and INCAT scores.

(A-C) display the correlations for the EMDA-CIDP patients' (n=43) maximum daily steps over the entire observational period. Notably, in panel (A), a fitted sigmoid function is included to illustrate a possible leveling-off effect at higher step counts, suggesting a potential ceiling effect in the relationship between maximum steps and I-RODS scores.

(D-F) show the correlations for the maximum daily steps during the last month of the EMDA-CIDP study period.

(G-I) illustrate the correlations for maximum daily steps over a 6-month period in the control real-world smartphone cohort (n=20).

(J-L) present the correlations for the last month's maximum daily steps within the same real-world cohort.

Overall, these plots demonstrate varying degrees of correlation between maximum daily steps and clinical severity, with generally similar trends between the EMDA-CIDP study cohort and the real-world control cohort. The strongest correlations are observed with I-RODS and INCAT scores, especially in the EMDA cohort, while correlations with the MRC sum score are generally weaker.

CIDP: Chronic Inflammatory Demyelinating Polyradiculoneuropathy, INCAT: Inflammatory Neuropathy Cause and Treatment, I-RODS: Inflammatory Rasch-built Overall Disability Scale, LOESS: Locally Estimated Scatterplot Smoothing, MRC: Medical Research Council, RW: Real-World.

Supplementary Figure 12: Scatter plots of maximum daily steps correlated with WHOQOL domains in CIDP patients and a real-world cohort

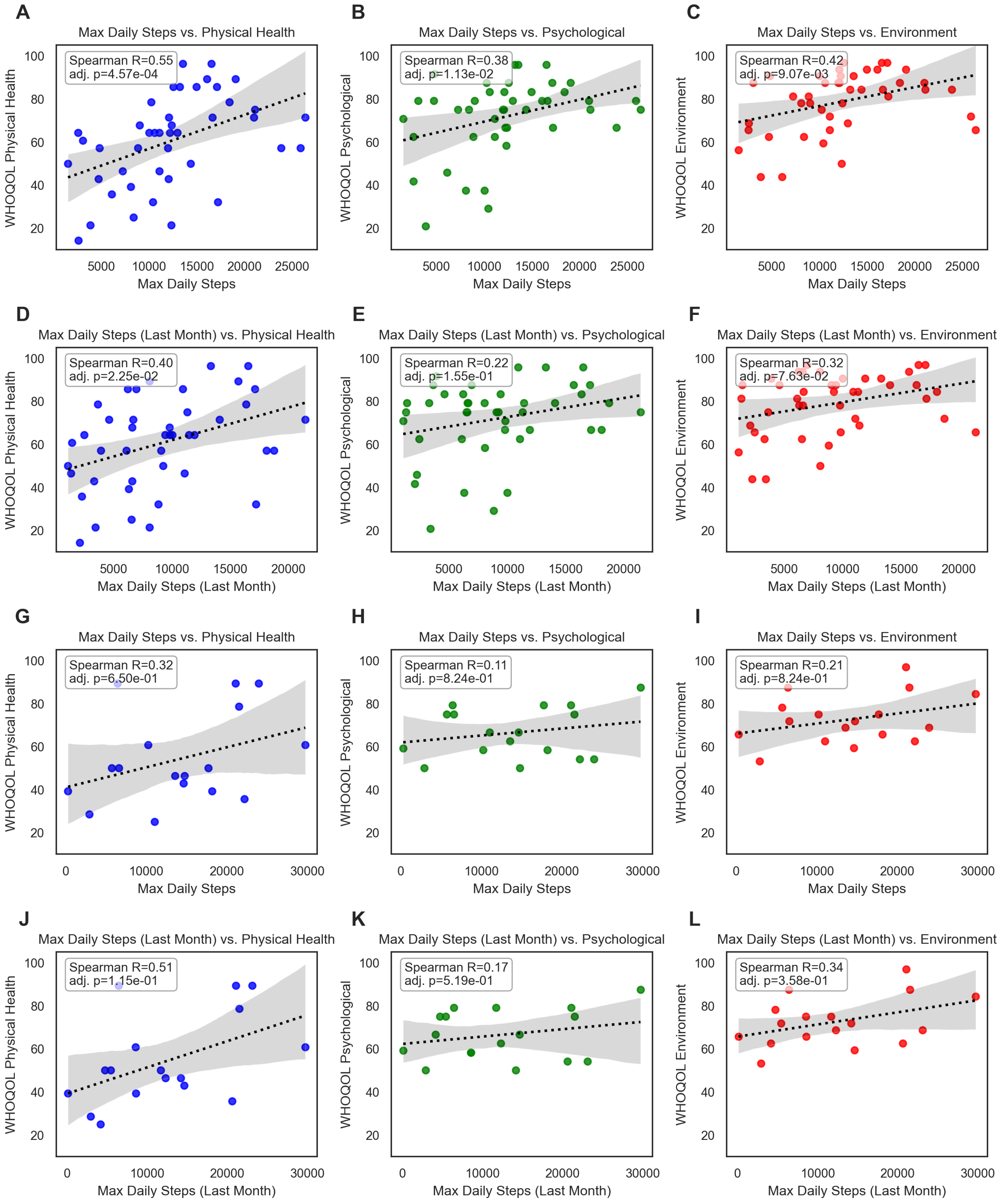

See next page for legend.

Supplementary Figure 12: Scatter plots of maximum daily steps correlated with WHOQOL domains in CIDP patients and a real-world cohort

This figure presents scatter plots illustrating the relationship between maximum daily steps and three WHOQOL domains: Physical Health (blue – left column), Psychological (green – middle column), and Environment (red – right column). The analysis is conducted across different observation periods and cohorts. All reported R values in the graphs are row-wise Holm-adjusted Spearman correlation coefficients, reflecting the rank-based relationship between maximum steps and the QOL results. The linear trendlines in these plots are for illustrative purposes only and should be interpreted as approximations, not as precise representations of the underlying relationships, especially given the complexity of QOL domains. An alternate representation using LOESS regression can be found in Supplementary Figure 13.

(A-C) display the correlations for the EMDA-CIDP patients' (n=43) maximum daily steps over the entire observational period.

(D-F) show the correlations for the maximum daily steps during the last month of the EMDA-CIDP study period.

(G-I) illustrate the correlations for maximum daily steps over a 6-month period in the control real-world smartphone cohort (n=17).

(J-L) present the correlations for the last month's maximum daily steps within the same real-world cohort.

Overall, these plots demonstrate varying degrees of correlation between maximum daily steps and QOL domains, with generally similar trends between the EMDA-CIDP study cohort and the real-world control cohort, although none of the analyses were significant after correction in the real-world smartphone cohort. The strongest correlations are observed with the Physical Health domain.

CIDP: Chronic Inflammatory Demyelinating Polyradiculoneuropathy, LOESS: Locally Estimated Scatterplot Smoothing, RW: Real-World, WHOQOL: World Health Organization Quality of Life.

Supplementary Figure 13: Scatter plots of maximum daily steps correlated with WHOQOL domains in CIDP patients and a real-world cohort using LOESS regression

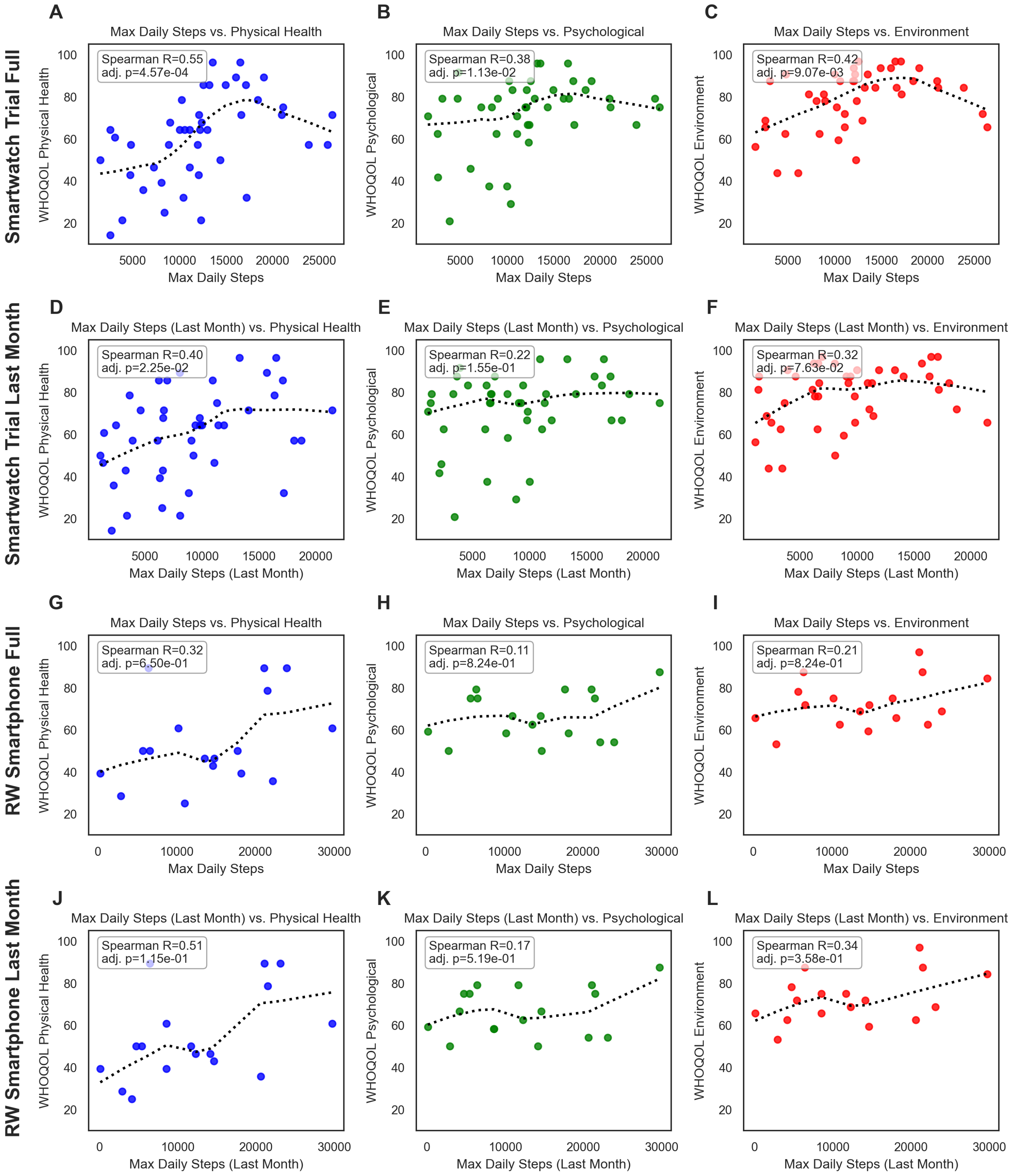

See next page for legend.

Supplementary Figure 13: Scatter plots of maximum daily steps correlated with WHOQOL domains in CIDP patients and a real-world cohort using LOESS regression

This figure presents scatter plots illustrating the relationship between maximum daily steps and three WHOQOL domains: Physical Health (blue – left column), Psychological (green – middle column), and Environment (red – right column). The analysis is conducted across different observation periods and cohorts. All reported R values in the graphs are row-wise Holm-adjusted Spearman correlation coefficients, reflecting the rank-based relationship between maximum steps and the QOL results. The LOESS regression trendlines in these plots provide a more technically accurate representation of the relationships, capturing potential non-linear trends that linear models may not adequately reflect, especially given the complexity of QOL domains.

(A-C) display the correlations for the EMDA-CIDP patients' (n=43) maximum daily steps over the entire observational period.

(D-F) show the correlations for the maximum daily steps during the last month of the EMDA-CIDP study period.

(G-I) illustrate the correlations for maximum daily steps over a 6-month period in the control real-world smartphone cohort (n=20).

(J-L) present the correlations for the last month's maximum daily steps within the same real-world cohort.

Overall, these plots demonstrate varying degrees of correlation between maximum daily steps and QOL domains, with generally similar trends between the EMDA-CIDP study cohort and the real-world control cohort, although none of the analyses were significant after correction in the real-world smartphone cohort. The strongest correlations are observed with the Physical Health domain.

CIDP: Chronic Inflammatory Demyelinating Polyradiculoneuropathy, LOESS: Locally Estimated Scatterplot Smoothing, RW: Real-World, WHOQOL: World Health Organization Quality of Life.

Supplementary Figure 14: Heatmap of spearman correlations between daytime/peak steps and clinical/QOL metrics in CIDP patients

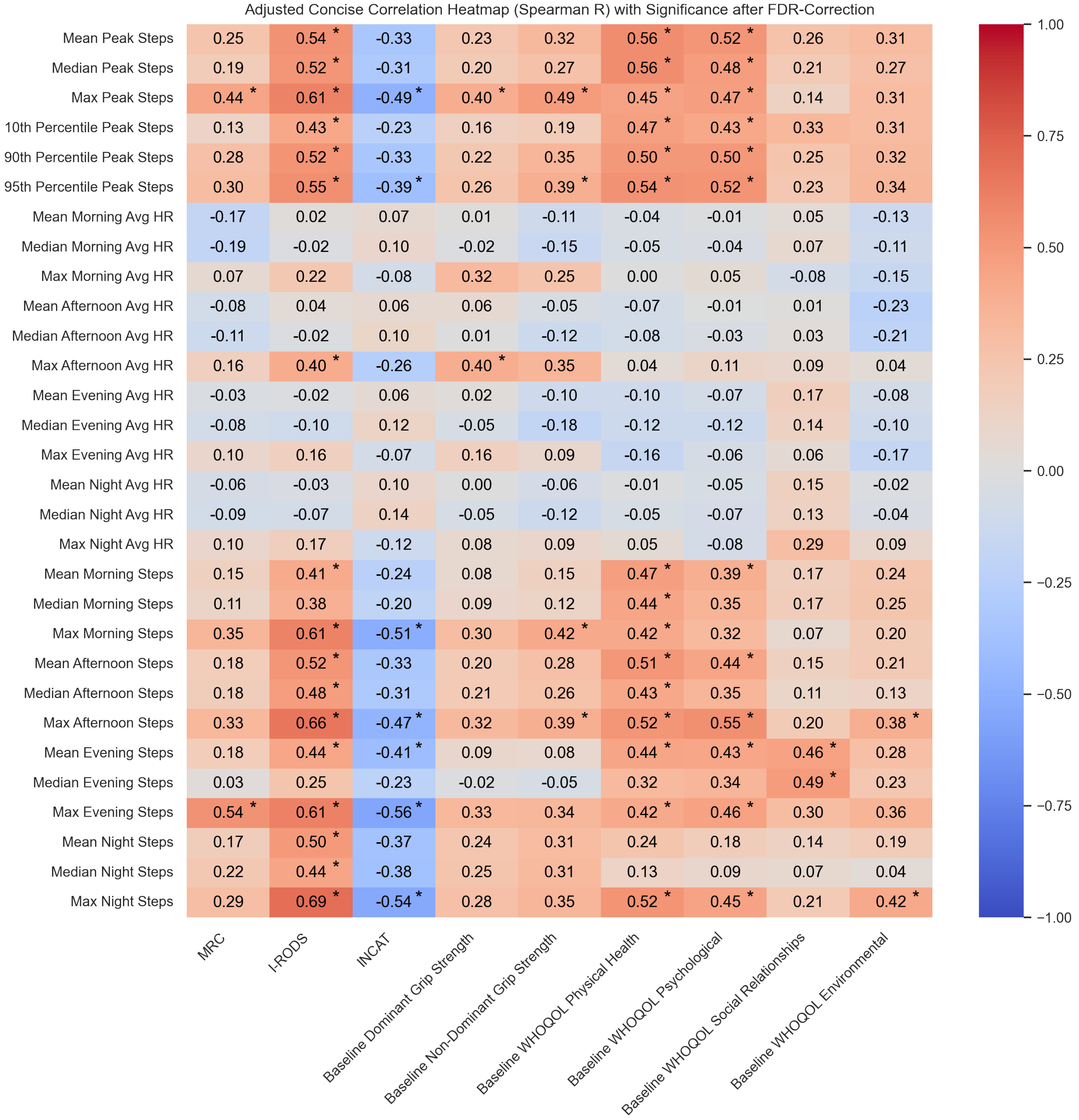

See next page for legend.

Supplementary Figure 14: Heatmap of spearman correlations between daytime/peak steps and clinical/QOL metrics in CIDP patients

This heatmap displays Spearman correlation coefficients between various stratifications of daily steps (by daytime periods and peak steps) and clinical as well as Quality of Life (QOL) metrics in CIDP patients. Peak steps are defined as the highest number of steps taken within a single hour each day, capturing time dynamics in physical activity. Significant correlations after FDR Benjamini-Hochberg correction are indicated with stars.

The analysis highlights interesting correlations, particularly with mean evening steps and maximum night steps showing notable relationships with QOL domains, suggesting the potential importance of these specific timeframes in patient well-being.

CIDP: Chronic Inflammatory Demyelinating Polyradiculoneuropathy , FDR: False Discovery Rate, INCAT: Inflammatory Neuropathy Cause and Treatment, I-RODS: Inflammatory Rasch-built Overall Disability Scale, MRC: Medical Research Council, QOL: Quality of Life, WHOQOL: World Health Organization Quality of Life.
